# Unveiling Clusters of RNA Transcript Pairs Associated with Markers of Alzheimer’s Disease Progression

**DOI:** 10.1371/journal.pone.0045535

**Published:** 2012-09-21

**Authors:** Ahmed Shamsul Arefin, Luke Mathieson, Daniel Johnstone, Regina Berretta, Pablo Moscato

**Affiliations:** 1 Centre for Bioinformatics, Biomarker Discovery and Information-Based Medicine, The University of Newcastle, Callaghan, New South Wales, Australia; 2 Faculty of Science, Macquarie University, Sydney, Australia; 3 Hunter Medical Research Institute, Information Based Medicine Program, Rankin Park, New South Wales, Australia; 4 Australian Research Council Centre of Excellence in Bioinformatics, Callaghan, New South Wales, Australia; Biological Research Centre of the Hungarian Academy of Sciences, Hungary

## Abstract

**Background:**

One primary goal of transcriptomic studies is identifying gene expression patterns correlating with disease progression. This is usually achieved by considering transcripts that independently pass an arbitrary threshold (e.g. *p*<0.05). In diseases involving severe perturbations of multiple molecular systems, such as Alzheimer’s disease (AD), this univariate approach often results in a large list of seemingly unrelated transcripts. We utilised a powerful multivariate clustering approach to identify clusters of RNA biomarkers strongly associated with markers of AD progression. We discuss the value of considering pairs of transcripts which, in contrast to individual transcripts, helps avoid natural human transcriptome variation that can overshadow disease-related changes.

**Methodology/Principal Findings:**

We re-analysed a dataset of hippocampal transcript levels in nine controls and 22 patients with varying degrees of AD. A large-scale clustering approach determined groups of transcript probe sets that correlate strongly with measures of AD progression, including both clinical and neuropathological measures and quantifiers of the characteristic transcriptome shift from control to severe AD. This enabled identification of restricted groups of highly correlated probe sets from an initial list of 1,372 previously published by our group. We repeated this analysis on an expanded dataset that included all pair-wise combinations of the 1,372 probe sets. As clustering of this massive dataset is unfeasible using standard computational tools, we adapted and re-implemented a clustering algorithm that uses external memory algorithmic approach. This identified various pairs that strongly correlated with markers of AD progression and highlighted important biological pathways potentially involved in AD pathogenesis.

**Conclusions/Significance:**

Our analyses demonstrate that, although there exists a relatively large molecular signature of AD progression, only a small number of transcripts recurrently cluster with different markers of AD progression. Furthermore, considering the relationship between two transcripts can highlight important biological relationships that are missed when considering either transcript in isolation.

## Introduction

Alzheimer’s disease (AD) is an irreversible brain disease that begins with mild memory impairment but eventually progresses to severe brain dysfunction and dementia. The prevalence of AD is rising dramatically due to an increasingly ageing population [1]. Early diagnosis is challenging as it can be difficult to discriminate the initial manifestations of the disease from cognitive decline that occurs as a function of normal aging [2]. Intensive efforts are being made to better understand AD and identify appropriate treatments, however the molecular mechanisms underlying the disease are still far from being understood.

In 2004, Blalock and colleagues [3] made an important contribution towards finding a set of molecular biomarkers that correlate with the progression of AD in one region of the brain. Using microarray technology, they assessed RNA transcript levels in post-mortem hippocampal tissue from 9 controls and 22 patients with varying degrees of AD severity. Participants were categorized, based primarily on Mini-Mental State Examination (MMSE) score [4], into one of four clinical groups: Control, Incipient AD, Moderate AD or Severe AD (see Materials and Methods for details of classification).

A total of 22,286 probe sets for protein coding RNA (i.e. mRNA) and non-coding RNA (ncRNA) were used to interrogate the transcriptome. After excluding probe sets with signals below detection thresholds and probe sets targeting unidentified transcripts (e.g. expressed sequence tags), Blalock and colleagues assessed the Pearson’s correlation of each of the remaining 9,921 probe set values with MMSE score and neurofibrillary tangle (NFT) count. This analysis revealed 3,413 genes that are significantly correlated (at *p*<0.05) with MMSE score, NFT count or both [3].

In 2010, Gomez Ravetti and colleagues (including two authors of this contribution) re-analyzed the publicly available dataset of Blalock using a different method, uncovering a 1,372-probe set signature that presents a remarkably high consensus with established phenotypic markers of AD progression [5]. Instead of just assessing correlations between gene expression and clinical measures of AD (such as MMSE score or NFT count), they employed an integrated approach based on combinatorial optimization techniques [6], [7] and Information Theory. Briefly, divergence of the gene expression profile of each individual sample from the average characteristic profile of the “Control” group was computed using the *Jensen-Shannon divergence* [8]. Similarly, this approach was used to compute the convergence of the gene expression profile of each individual sample to the average characteristic profile of the “Severe AD” group. This allowed the authors to identify genes with expression levels that correlate with the characteristic molecular progression from normal cognition to severe AD. In the current report, we use these quantifiers (which we will simply term as *JSD_control_* and *JSD_severe_*, respectively) as measures of AD progression, in addition to three common phenotypic markers (MMSE score, NFT count and Braak staging; obtained from [3]). We will refer collectively to these measures of AD progression as ‘progression markers’.

The main objective of this work is to identify a *reduced set of pairs of RNA transcripts* that strongly cluster with markers of AD progression. The analysis of these clusters will also help to identify individual transcripts that recurrently appear in many pairs, and may thus guide the selection of candidate molecules for further research. Towards this end, we apply a large-scale graph-based clustering approach to datasets derived from the 1,372-probe set signature identified by Gomez Ravetti et al. [5] to identify molecular features that correlate with the different ‘progression markers’. These datasets include the original 1,372-probe set signature, all pair-wise ratios that can be computed from the 1,372-probe set signature and an expanded dataset containing all pair-wise differences, summations, ratios and products that can be computed from the original signature (giving a total of 3,762,024 probe set combinations). We will refer to these probe set pairs as ‘metafeatures’ [9].

To cluster such a large number of metafeatures, we re-implemented and enhanced the MST*k*NN algorithm in [10] by using the external memory (EM) approaches in [11], [12], [13]. External memory algorithms are known for their efficiency in handling large-scale data sets [14], [15], [16] and the MST*k*NN algorithm is a graph based data clustering algorithm that has successfully been applied in several applications including the analysis of stock market time series [17], a gene expression dataset [18], a prostate cancer trial dataset [19] and has been integrated with a combinatorial optimization based graph visualization layout [20]. Our previous work indicates that the MST*k*NN algorithm produces meaningful clusters (see [17], [18], [20]) and our proposed modification to this algorithm is still capable of producing reasonable clustering structure in terms of homogeneity and separation (Table 1).

**Table 1:** Performance comparison of the EM MST*k*NN with *k*-Means, SOM, CLICK and the original MST*k*NN algorithms, in terms of homogeneity and separation.

Data	Methods/Algorithm	Parameter	H_avg_	S_avg_	#Clusters	Time
AD Signature data set(*n* = 1,372)	*k*-Means	*k* = 5	0.179	0.121	5	<0.5 min
		*k* = 120	0.394	0.172	120	<1 min
	SOM	2X5 grid	0.185	0.183	6	<0.5min
		5X5 grid	0.217	0.142	14	<1 min
	CLICK	–	0.606	0.245	5	<1 min
	MSTkNN	–	0.780	0.369	226	<0.5 min
	EM MST*k*NN(this paper)	–	0.789	0.370	228	<0.2 min
AD ratios data set(*n* = 941,885)	*k*-Means, SOM,CLICK, MST*k*NN	–	Not Available	Not Available	Not Available	Not Available
	EM MST*k*NN(this paper)	–	0.812	0.420	40,139	30 min
AD ratios- sums-diffs-prods dataset(*n* = 3,763,403)	EM MST*k*NN(this paper)	–	0.879	0.521	121,611	120 min

The implementations of the *k*-Means, SOM, CLICK algorithms are obtained from the Expander microarray data cluster tool in [124]. The homogeneity and separation are computed using the definition in [124]. The AD ratio metafeatures data set is generated by taking pair-wise ratios between the features in 1,372-probe AD signatures [5] and including MMSE score, NFT count, Braak staging, *JSD_control_* and *JSD_severe_* as five progression markers. The other data set contains four different types of metafeatures (ratios, summations, differences and products) and the aforementioned progression markers.

After clustering all the metafeatures together with the different progression markers, we attempt to uncover pairs of probe sets that jointly cluster with each progression marker. Additionally, we identify some probe sets in these pairs that not only cluster with different progression markers but also relate to genes that share common biological pathways. We annotate these pairs of markers with the most recent information available. We also look at the expression of some of these markers in a different transcriptomic study that involves several regions of the brain in search for consensus among different studies.

## Results

Results for each of the datasets are presented in the following order: 1. composition of the clusters containing the aforementioned progression markers, 2. results of functional analyses carried out using publicly available tools (iHop, Gather, GIM) and 3. a bibliographic (Pubmed) characterization of certain highlighted probe sets. Probe sets are highlighted for several reasons, including having a strong correlation with one or more progression markers or appearing in a metafeature with a probe set for another gene which has a potential role in AD or in other brain disease. The clustering outcomes are given in File S1, File S2 and File S3. Where a particular probe set has been highlighted and discussed in our previous publication on this dataset [5], we refer the reader to this paper rather than re-iterating discussion points in the present report.

### 1,372-probe set signature (File S1)

**Figure 1:**
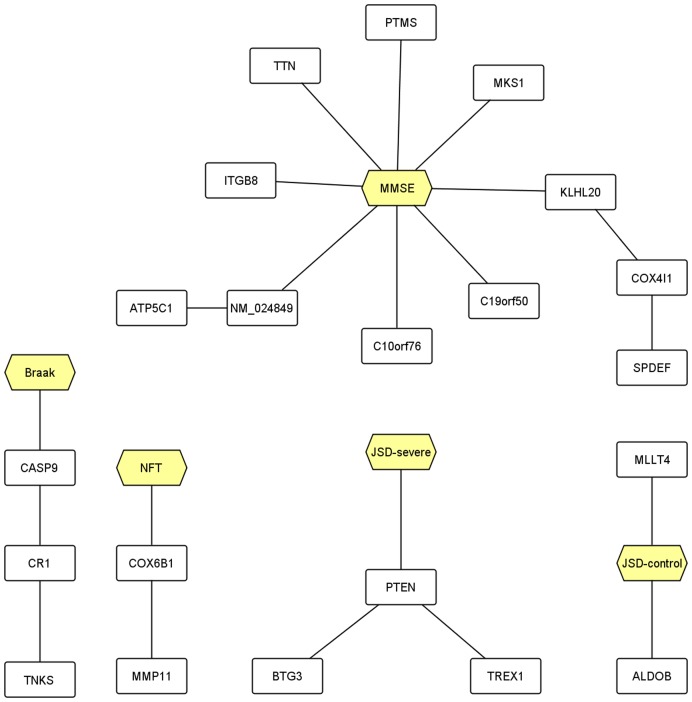
Visualization of the clustering outcome of the 1372-probe set signature. The figure shows only the clusters that contain the progression markers (hexagonal nodes). We note that the probe set for *PTEN*, whose product has been recently observed to localize with intracellular NFTs [36], has values that correlate strongly with the Jensen-Shannon divergence of the severe profile (*JSD_severe_*).

First, we analysed the 1,372-probe set signature from [5] using the proposed clustering algorithm and found a total of 228 clusters. We then identified the clusters that contain the proposed progression markers (Figure 1 and Table 2).

**Table 2:** Clustering outcomes for the 1372-probe set signature.

Progression Marker	Gene Symbol. Probe Set ID	Correlation Coefficient	KEGG Pathway
**MMSE**	ATP5C1. 213366_x_at	0.764201	ATP synthesis
	COX4I1. 202698_x_at	0.590462	Oxidative phosphorylation
	**SPDEF.** 214403_x_at	−0.48268	
	**MKS1.** 218630_at	−0.69599	
	**PTMS.** 218044_x_at	−0.72683	
	**TTN.** 208195_at	−0.73718	Calcium signaling pathway, Focal adhesion
	**C19orf50.** 200076_s_at	−0.80389	
	KLHL20. 204177_s_at	−0.81714	
	C10orf76. 55662_at	−0.82583	
	ITGB8. 205816_at	−0.86993	ECM-receptor interaction, Focal adhesion
	**NM_024849.** 220531_at	−0.88067	
**NFT**	COX6B1. 201441_at	0.564201	Oxidative phosphorylation
	MMP11. 203876_s_at	−0.290462	
**Braak**	**CR1.** 206244_at	0.363241	Complement and coagulation cascades
	CASP9. 210775_x_at	−0.240432	Apoptosis, MAPK signaling pathway
	**TNKS.** 216695_s_at	−0.287898	
**JSD_control_**	**MLLT4.** 208512_s_at	0.723242	Adherens junction, Tight junction
	**ALDOB.** 217238_s_at	0.480468	Carbon fixation, Glycolysis
**JSD_severe_**	**BTG3.** 215425_at	0.464201	ATP synthesis
	**PTEN.** 211711_s_at	0.250462	Phosphatidylinositol signaling system, Tight junction
	**TREX1.** 34689_at	0.236458	

For each progression marker, probe sets have been ordered according to their Spearman’s rank correlation with the progression marker. Gene symbols in boldface indicate that they were previously discussed in [5] and gene symbols with underlined boldface represent the cases for which a putative relationship exists in the published literature between the gene and AD.

#### Cluster containing MMSE score

A total of 11 probe sets clustered with *MMSE score*. Among the gene transcripts targeted by these probe sets, *TTN* (Titin/Connectin) may have a role in AD progression through its ability to form amyloid aggregates [21]. The genes *ATP5C1*, *COX4I1*, *KLHL20*, *ITGB8* and *C10orf76* were highlighted by the analysis in Gomez Ravetti et al. [5] and we refer the reader to this paper for extensive discussion of these genes in the context of AD. We identified several probe sets that uniquely cluster with *MMSE score* and target transcripts for genes not previously associated with AD. These included *PTMS* (parathymosin), a chromatin remodelling protein essential for cell cycle progression and proliferation of normal and malignant cells, *MKS1* (Meckel syndrome, type 1), mutations in which are associated with a malformation of central nervous system known as Meckel syndrome, and *SPDEF* and a probe set for *C19orf50*.

#### Cluster containing NFT count

Two probe sets clustered with *NFT count*. These probe sets targeted transcripts for *COX6B1* and *MMP11*, both of which were highlighted in Gomez Ravetti et al. [5] and will not be discussed in detail here.

#### Cluster containing braak staging

Three probe sets clustered with the *Braak staging* values. These probe sets targeted transcripts for *CASP9* (caspase 9), which was previously highlighted by the analysis in Gomez Ravetti et al. [5], *TNKS* (tankyrase) and *CR1* (complement receptor 1), which is genetically associated with the risk of AD [22], [23], [24], [25], [26], [27], [28], [29], [30] and entorhinal cortex volume in young healthy adults [31] (see also [32]).

#### Clusters containing JSD_control_ and JSD_severe_

Two probe sets clustered with *JSD_control_* - these related to the genes *ALDOB* (aldolase B, fructose-bisphosphate) and *MLLT4* (myeloid/lymphoid or mixed-lineage leukemia, translocated to, 4), which is reportedly involved in the formation and remodelling of synapses in hippocampus [33]. Three probe sets clustered with *JSD_severe_*. These related to the genes *BTG3* (BTG family, member 3), which is related to prognosis of neuropathic symptoms in paraneoplastic neuropathy [34], *TREX1* (three prime repair exonuclease 1), mutations in which can cause a neurovascular disorder involving progressive cognitive decline due to brain degeneration [35], and *PTEN* (phosphatase and tensin homolog). Altered distribution of *PTEN* has been reported in degenerating neurons in AD; specifically, a delocalization from the nucleus to the cytoplasm and accumulation in intracellular neurofibrillary tangles [36].

#### Comparing clustering outcomes with statistics-based outcomes

An alternative method for selecting probe sets highly correlated with progression markers is to simply perform regression analysis and identify the probe sets with the highest correlation coefficient or lowest *p*-value. In essence this is a univariate approach, where the selection of a particular probe set is independent of which other probe sets are selected. In contrast, the MST*k*NN-based clustering method used here is a multivariate approach which considers the interrelationships of different probe sets in its selection. As such, we would expect the MST*k*NN-based method to identify features of potential biological relevance that are missed by univariate approaches.

To investigate the similarities and differences in outcomes of multivariate and univariate approaches, we compared the probe sets identified by our clustering method with those having the highest, most significant correlations by regression analysis (see Figure S1). For example, we compared the 11 probe sets clustered with MMSE to the 11 probe sets most significantly correlated with MMSE by regression analysis. While there were some similarities in the probe sets identified, there were also important differences. Notably, some genes of particular relevance to AD were selected by the clustering method but not by filtering based on statistical significance. For example, clustering with Braak staging identified a probe set targeting CR1 which, as mentioned above, is widely proposed as a genetic risk factor for AD, however this probe set was not identified by the statistical method.

### 941,885 Ratio Metafeatures Data Set (File S2)

Next, we conducted a cluster analysis using the proposed external memory clustering algorithm on the 941,885 metafeatures generated by calculating the *ratio* of each pair of probe sets from the 1,372-probe set signature [5]. There were a total of 40,139 clusters in the data set, from which we again identified the clusters that contain the previously mentioned progression markers.

#### Cluster containing MMSE score

A total of 32 *ratio* metafeatures (containing 35 different probe sets) clustered with *MMSE score* (Table 3). The cluster contained metafeatures involving probe sets for five genes previously studied in the context of AD (*TTN* [37], [38], *PPIA* [39], [40],[41], *VSNL1* [42], [43], [44], [45], [46], [47], [48], *NEFL* [49], [50], [51] and *FXYD6* [52]). We also identified, in different clustered metafeatures, probe sets for 15 other genes (*FOXO1*, *ACACA*, *CASK*, *PTEN*, *PTN*, *ATP5C1*, *LDHA*, *PRKAR2B*, *CPT2*, *DDX1*, *ITGBL1*, *TCF7L2*, *C10orf76*, the non-coding RNA *TUG1* and *SCFD1*) previously highlighted in the analysis of Gomez Ravetti et al. [5]. Additionally, we found 12 metafeatures in which both of the probe sets comprising the metafeature relate to genes in a common pathway (Table 3). These gene pairs were *PRKCB1*/*PTEN* (phosphatidylinositol signaling system), *FOXO1*/*ACACA* and *PRKAR2B*/*ACACA* (insulin signaling pathway), *ACACA*/*LDHA* (propanoate metabolism), *COX6A1*/*ATP5C1* (oxidative phosphorylation), *TCF7L2*/*ACTN1* (adherens junction), *CASK*/*PTEN* (tight junction), *C10orf76*/*PPIA* (ECM-receptor interaction), *ACTN1*/*VCL*, *ITGB8*/*TTN*, *ITGB8*/*PTN* and *TTN*/*PRKCB1* (focal adhesion).

**Table 3:** Ratio metafeatures clustered with MMSE score.

Metafeature (Gene Symbol. Probe Set ID)	Correlation Coefficient	Common KEGG Pathways
**PPIA**.212661_x_at/C3orf60.209177_at	0.884365	
PIGO.209998_at/**CASK**.211208_s_at	0.760958	
PURA.213806_at/AJ225093.211835_at	0.673877	
**PRKCB1**.209685_s_at/**PTEN**.222176_at	−0.68675	Phosphatidylinositol signaling system
**FOXO1**.202724_s_at/**ACACA**.212186_at	−0.78654	Insulin signaling pathway
**CASK**.211208_s_at/**PTEN**.222176_at	−0.83565	Tight junction
RPL23A.203012_x_at/**ATP5C1**.213366_x_at	−0.83758	
**CASK**.211208_s_at/**ATP5C1**.213366_x_at	−0.86355	
**ACACA**.212186_at/**LDHA**.200650_s_at	−0.87642	Propanoate metabolism
**PRKAR2B**.203680_at/**ACACA**.212186_at	−0.88001	Insulin signaling pathway
COX6A1.200925_at/**ATP5C1**.213366_x_at	−0.88233	Oxidative phosphorylation
RPL23A.203012_x_at/**PPIA**.211765_x_at	−0.88328	
ACTN1.208636_at/VCL.200930_s_at	−0.88344	Focal adhesion
**ITGB8**.205816_at/**TTN**.208195_at	−0.88642	Focal adhesion
**ITGB8**.205816_at/PTN.209466_x_at	−0.88642	Focal adhesion
**CPT2**.204264_at/**ATP5C1**.213366_x_at	−0.89851	
**TTN**.208195_at/**PRKCB1**.209685_s_at	−0.90065	Focal adhesion
SDC1.201287_s_at/**DDX1**.201241_at	−0.90504	
**TTN**.208195_at/**NEFL**.221805_at	−0.91592	
ZNF34.219801_at/**FXYD6**.217897_at	−0.9181	
**AL359052**.214927_at/**ATP5C1**.205711_x_at	−0.9188	
NUCKS1.217802_s_at/**PPIA**.212661_x_at	−0.91944	
**TCF7L2**.212761_at/ACTN1.208636_at	−0.92353	Adherens junction
**TTN**.208195_at/**VSNL1**.203798_s_at	−0.93006	
**CASK**.211208_s_at/**PTN**.209466_x_at	−0.9301	
NM_024849.220531_at/MRPL16.217980_s_at	−0.93682	
AJ225093.211835_at/**ATP5C1**.205711_x_at	−0.93986	
**C10orf76**.55662_at/**PPIA**.212661_x_at	−0.94211	
**ITGB8**.205816_at/SDC1.201287_s_at	−0.95719	ECM-receptor interaction
**ITGB8**.205816_at/**PPIA**.212661_x_at	−0.95911	
**CASK**.211208_s_at/**PPIA**.211765_x_at	−0.95641	
TUG1.222244_s_at/**SCFD1**.215548_s_at	−0.95643	

Metafeatures are ordered by Spearman’s rank correlation with MMSE score. Genes in boldface indicate that they were previously discussed in [5] and genes with underlined boldface represent the cases for which the gene has been discussed in the context of AD in the published literature.

#### Cluster containing NFT count

Four *ratio* metafeatures (containing 8 different probe sets) clustered with *NFT count* (Table 4). The probe sets comprising these metafeatures did not target genes in common pathways but four of them targeted genes previously highlighted in the analysis of Gomez Ravetti et al. [5]: *ICA1*, *COX6B1*, *ATP5C1* and *ITGB8*.

**Table 4:** Ratio metafeatures clustered with NFT count.

Metafeature(Gene Symbol.Probe Set ID)	Correlation Coefficient
**ICA1.207949_s_at**/RUNX2.216994_s_at	−0.78797
TSPAN9.220968_s_at/**NRG1**.206343_s_at	−0.87831
PTMS.218044_x_at/GRPEL1.212434_at	−0.87839
**ITGB8**.205816_at/ABAT.209460_at	−0.87857

Metafeatures are ordered by Spearman’s rank correlation with NFT count. Genes in boldface indicate that they were previously discussed in [5] and genes with underlined boldface represent the cases for which the gene has been discussed in the context of AD in the published literature.

#### Cluster containing braak staging

A total of 27 *ratio* metafeatures clustered with *Braak staging* values (Table 5). Three of these were comprised of probe sets targeting genes that can be mapped to common KEGG pathways: *CR1*/*SERPINA5* (complement and coagulation cascades), *MDH2*/*ACO2* (TCA cycle) and *ITGB5*/*TNXB* (ECM-receptor interaction). Of the various probe sets identified in this cluster, only one targets a gene previously implicated in AD - *CR1* (discussed above). Metafeatures in the cluster also contained probe sets targeting six other genes previously highlighted by the analysis of Gomez Ravetti et al. [5]: *PTN*, *PPT1*, *RIMS2*, *ASTN1*, *ITGB5* and *PAX6*. Many of the metafeatures that showed a positive correlation with Braak staging were dominated by a probe set for *LRRC48* (leucine rich repeat containing 48), a gene which is currently poorly characterized.

**Table 5:** Ratio metafeatures clustered with Braak staging.

Metafeature (Gene Symbol. Probe Set ID)	Correlation Coefficient	Common KEGG Pathways
LRRC48.208140_s_at/SLC6A1.205152_at	0.885391	
LRRC48.208140_s_at/CHRDL1.209763_at	0.875476	
LRRC48.208140_s_at/**PTN**.209466_x_at	0.868268	
LRRC48.208140_s_at/PTPN4.205171_at	0.853365	
LRRC48.208140_s_at/CAST.212586_at	0.847157	
LRRC48.208140_s_at/MAOA.212741_at	0.837757	
LRRC48.208140_s_at/**PPT1**.200975_at	0.837151	
C16orf57.218060_s_at/**RIMS2.**206137_at	0.832873	
LRRC48.208140_s_at/ACO2.200793_s_at	0.830536	
LRRC48.208140_s_at/TIMM23.218118_s_at	0.828653	
LRRC48.208140_s_at/**ASTN1**.213197_at	0.815831	
PTTG1IP.200677_at/RANBP9.202583_s_at	0.786333	
CSF2RA.211286_x_at/BBS4.212745_s_at	0.775829	
LRRC48.208140_s_at/GABBR1.203146_s_at	0.761019	
LRRC48.208140_s_at/PRC1.218009_s_at	0.748013	
TRA@.209671_x_at/NKX3-1.209706_at	0.673362	
RHOQ.212122_at/TBCE.203715_at	0.668935	
**ITGB5**.201125_s_at/IFNGR2.201642_at	0.591936	
LRRC48.208140_s_at/SLC6A1.205152_at	−0.46804	
**CR1**.206244_at/SERPINA5.209443_at	−0.66797	Complement and coagulation cascades
AL520908.217833_at/LRRC48.208140_s_at	−0.68605	
MDH2.213333_at/ACO2.200793_s_at	−0.76804	TCA cycle
**PAX6**.205646_s_at/LRRC48.208140_s_at	−0.77464	
SFN.33322_i_at/LRRC48.208140_s_at	−0.78278	
BMX.206464_at/RGS3.220300_at	−0.82257	
**ITGB5**.201125_s_at/TNXB.216333_x_at	−0.86804	ECM-receptor interaction
C15orf39.204494_s_at/LRRC48.208140_s_at	−0.89186	

Metafeatures are ordered by Spearman’s rank correlation with Braak staging. Genes in boldface indicate that they were previously discussed in [5] and genes with underlined boldface represent the cases for which the gene has been discussed in the context of AD in the published literature. Most of the positively correlated metafeatures in this cluster are dominated by LRRC48 (See File S2 for details).

#### Cluster containing JSD_control_

A total of 93 *ratio* metafeatures (containing a total of 94 different probe sets) clustered with *JSD_control_* (Table 6), see also File S2 for complete list). Three of these metafeatures comprised probe sets targeting genes in common KEGG metabolic pathways: *LDHA*/*GOT2* (cysteine metabolism), *NDUFA10*/*ATP5C1* (oxidative phosphorylation) and *ATP5C1*/*ATP5H* (ATP synthesis). The probe sets contained in the clustered metafeatures targeted six genes that have previously been studied in the context of AD: *PPP2CA* *[53]*, [54], [55], *[56]*, *SERPINI1* [57], [58], [59], [60], [61], *OPA1* [62], [63], [64], [65], [66], *PPIA* and *CSF1* [67], [68], [69], [70], [71], [72]. Additionally, we identified probe sets targeting 27 other genes previously highlighted by the analysis of Gomez Ravetti et al. [5]: *NUFIP1*, *ATP6V1D*, *UQCRQ*, *DDX1*, *WASF1*, *ATP5C1*, *COX4I1*, *SNRK*, *PPP3CA*, *LDB2*, *COX7AP2*, *LAMTOR2*, *LDHA*, *PBX1*, *CAPRIN2*, *SLC25A6*, *SCFD1*, *DOPEY1*, *CSPG5*, *TUBG2*, *NRXN1*, *CADPS2*, *CRYM*, *FZD5*, *MAPK1*, *CASP9*, *PTN* and *ICA1*. Most of the metafeatures in this cluster that showed a positive correlation with the divergence from control to severe AD were dominated by *KLK3* (kallikrein 3), also known as prostate specific antigen, a well-known blood biomarker of prostate cancer [73]. To determine whether correlations involving *KLK3* levels were influenced by gender, we stratified our dataset by gender. We performed the clustering again in both gender-specific datasets and found that *KLK3* was completely absent in the same cluster of the dataset comprising only females. We therefore suggest that further study of *KLK3* in relation to AD should be done in male patients only.

**Table 6:** Ratio metafeatures clustered with *JSD_control_.*

Metafeature (Gene Symbol. Probe Set ID)	Correlation Coefficient	Common KEGG Pathways
KLK3.204582_s_at/MAST3.213045_at	0.852532	
KLK3.204582_s_at/**NUFIP1**.205136_s_at	0.792759	
KLK3.204582_s_at/ATP5H.210149_s_at	0.79046	
KLK3.204582_s_at/AW242701.213411_at	0.781264	
KLK3.204582_s_at/GOT2.200708_at	0.774367	
KLK3.204582_s_at/**KIAA1467**.213234_at	0.769769	
KLK3.204582_s_at/FMO5.205776_at	0.758274	
KLK3.204582_s_at/MDH2.213333_at	0.758067	
KLK3.204582_s_at/**PPP2CA**.208652_at	0.757663	
KLK3.204582_s_at/**ATP6V1D**.208898_at	0.744079	
**MAPK1**.208351_s_at/KLK3.204582_s_at	−0.71785	
**LDHA**.200650_s_at/GOT2.200708_at	−0.72409	Cysteine metabolism
**CASP9**.210775_x_at/KLK3.204582_s_at	−0.72609	
DNAJA4.220395_at/KLK3.204582_s_at	−0.74448	
**ICA1**.207949_s_at/**CSF1**.211839_s_at	−0.75766	
MDH2.213333_at/GOT2.200708_at	−0.77348	
TRIM26.202702_at/KLK3.204582_s_at	−0.84302	
AKR1B1.201272_at/KLK3.204582_s_at	−0.86002	
NDUFA10.217860_at/**ATP5C1**.205711_x_at	−0.86735	Oxidative phosphorylation
**ATP5C1**.205711_x_at/ATP5H.210149_s_at	−0.87348	ATP synthesis

We have selected 20 metafeatures (10 most positively correlated and 10 most negatively correlated) clustered with *JSD_control_* and ordered them by Spearman’s rank correlation with *JSD_control_*. Genes in boldface indicate that they were previously discussed in [5] and genes with underlined boldface represent the cases for which the gene has been discussed in the context of AD in the published literature. Most of the positively correlated metafeatures in this cluster are dominated by KLK3 (kallikrein 3) (See File S2 for details).

#### Cluster containing JSD_severe_

Nine *ratio* metafeatures clustered with *JSD_severe_* (Table 7). For four of these metafeatures, both probe sets comprising the metafeature targeted genes that can be mapped to a common KEGG pathway. These metafeatures were *MLLT4*/*PTEN* (tight junction), *PRKCB1*/*ATP2B2* (calcium signaling pathway), *TGFB2*/*PPP2CA* (TGF-β signaling pathway), *CYP3A4*/*CPT2* (fatty acid metabolism). Metafeatures in this cluster contained probe sets for three genes previously investigated in the context of AD (*CYP3A4* [74], [75], [76], *ATP2B2* [77], [78] and *PPP2CA*) and four other genes previously highlighted by the analysis of Gomez Ravetti et al. [5] (*PTEN*, *PRKCB1*, *FCAR* and *CPT2*).

**Table 7:** Ratio metafeatures clustered with *JSD_severe_.*

Metafeature (Gene Symbol. Probe Set ID)	Correlation Coefficient	Common KEGG Pathways
MLLT4.208512_s_at/**PTEN**.211711_s_at	0.842239	Tight junction
**CYP3A4**.205998_x_at/**PTEN**.211711_s_at	0.777368	
MLLT4.208512_s_at/CCDC6.204716_at	0.753547	
**PRKCB1**.209685_s_at/**ATP2B2**.204685_s_at	−0.2207	Calcium signaling pathway
CPNE3.202118_s_at/AL520908.217833_at	−0.46933	
TGFB2.209909_s_at/**PPP2CA**.208652_at	−0.67022	TGF-beta signaling pathway
**FCAR**.211307_s_at/AF043586.216394_x_at	−0.78256	
**CYP3A4**.205998_x_a/**CPT2**.204264_at	−0.87018	Fatty acid metabolism
N25732.204131_s_at/AF043586.216394_x_at	−0.9031	

Metafeatures are ordered by Spearman’s rank correlation with *JSD_severe_*. Genes in boldface indicate that they were previously discussed in [5] and genes with underlined boldface represent the cases for which the gene has been discussed in the context of AD in the published literature.

### Estimation of False Discovery Rate

Investigating a very large data space, such as that occupied by the many possible metafeatures, will inevitably lead to a number of false positive findings. In order to estimate the false discovery rate at different correlation coefficient thresholds, and therefore demonstrate the validity of our approach in identifying more than just random events, we performed a simple a *Monte-Carlo permutation test* by randomly permuting the MMSE scores of the 17 samples and computing the correlation of each metafeatures with the permuted MMSE labels. The results after 1,000 permutations reveal that, at all thresholds tested, there is clearly a higher number of strongly correlated metafeatures among our ratio metafeatures dataset than would be expected by chance alone (see Figure S2, Figure S3, Figure S4 and Figure S5).

### 3,763,403 *Ratio-sum-difference-product* Metafeatures Data Set (File S3)

We next applied our clustering algorithm to a data set of 3,763,403 metafeatures. This dataset was produced by calculating all pair-wise *differences*, *summations*, *ratios* and *products* of the 1,372-probes identified in Gomez Ravetti et al. [5].

The algorithm created a total of 121,611 clusters for the data set. We identified one larger cluster containing all of the progression markers. Due to the large number of metafeatures in the cluster, we focused only on the metafeatures with the strongest positive and negative correlations with each of the progression markers. We refer to File S3 for the details of this cluster.

#### Metafeatures correlating with MMSE Score

From the list of 50 metafeatures most strongly correlated (25 positively and 25 negatively) with *MMSE score*, we identified five that involve probe sets that target genes in common KEGG pathways (Table 8). This list of metafeatures also involved probe sets targeting genes previously investigated in the context of AD (*PPIA*, *TTN, FXYD6*, *VSNL1*, *SERPINI1*(see above), *PLCB1* [79], [80], [81], *IL15* [82], [83], [84], *NRG1* [85], [86], [87], *SERTAD2* [88]) and genes highlighted by the analysis in Ravetti et al. [5] (*ICA1*, *ITGB8*, *GABBR2*, *CSPG5*, *ATP2B2*, *C10orf76*, *PRKAR2B*, *ACACA*, *MYT1L*, *KLHL20*, *PTEN*, *LDHA*, *AFF1*, *TUG1*, *RBM19*, *CPT2*, *ZBTB20*, *ITGBL1*). We refer the reader to the paper of Gomez Ravetti et al. [5] for discussion of these genes in the context of AD.

**Table 8:** Ratio-sum-difference-product metafeatures clustered with MMSE score.

Metafeature (Gene Symbol. Probe Set ID)	Correlation Coefficient	Common KEGG Pathways
**ICA1**.207949_s_at-**ITGB8**.205816_at	0.940945	
**GABBR2**.209990_s_at+**PPIA**.212661_x_at	0.9268	
LTBP1.202728_s_at-**ITGB8**.205816_at	0.923973	
RRAD.204803_s_at-**ITGB8**.205816_at	0.922066	
**CSPG5**.39966_at*ATP5C1.213366_x_at	0.917444	
**ATP5C1**.213366_x_at*FXYD6.217897_at	0.911438	
MKL2.218259_at+**PPIA**.212661_x_at	0.909957	
**PPIA**.212661_x_at+**ATP2B2**.204685_s_at	0.908852	
**ATP5C1**.213366_x_at+KIAA1107.214098_at	0.903328	
**ICA1**.207949_s_at-**C10orf76**.55662_at	0.902256	
**PPIA**.212661_x_at+KCNQ2.205737_at	0.900168	
**PRKAR2B**.203680_at*ACACA.212186_at	0.8986	Insulin signaling pathway
**ATP5C1**.205711_x_at*FXYD6.217897_at	0.898298	
VCL.200930_s_at-**C10orf76**.55662_at	0.897422	
**PPIA**.212661_x_at+ARF3.200734_s_at	0.895376	
RIBC2.206526_at-**ITGB8**.205816_at	0.894556	
**CSPG5**.39966_at+**ATP5C1**.213366_x_at	0.894256	
AW851559.216056_at-**ZBTB20**.222357_at	0.893367	
**MYT1L**.210016_at+**PPIA**.212661_x_at	0.893136	
**PPIA**.212661_x_at+AW514267.214945_at	0.892278	
RRAD.204803_s_at-**KLHL20**.204177_s_at	0.891174	
**PTEN**.222176_at*TTN.208195_at	0.891049	Focal adhesion
**ATP5C1**.213366_x_at+**FXYD6**.217897_at	0.890893	
**AI708767**.211978_x_at*FXYD6.217897_at	0.890212	
**ATP5C1**.213366_x_at*ACACA.212186_at	0.888843	
U82303.216702_x_at+NM_018601.220880_at	−0.90422	
U82303.216702_x_at+**PTEN**.211711_s_at	−0.90498	
**ITGB8**.205816_at*SDC1.201287_s_at	−0.90504	ECM-receptor interaction
ZNF34.219801_at-**NRG1**.206343_s_at	−0.90504	
U82303.216702_x_at+**SERTAD2**.202656_s_at	−0.90546	
JPH2.220385_at+NM_024849.220531_at	−0.90598	
**ZBTB20**.222357_at-**NRG1**.206343_s_at	−0.90613	
U82303.216702_x_at+**RBM19**.205115_s_at	−0.90613	
PTMS.218044_x_at+NM_024849.220531_at	−0.90683	
U82303.216702_x_at+TPP1.214195_at	−0.90775	
**KLHL20**.204177_s_at+**AL359052**.214927_at	−0.90825	
**ITGB8**.205816_at+**TTN**.208195_at	−0.90941	Focal adhesion
**KLHL20**.204177_s_at+UMOD.206716_at	−0.90994	
TSPAN9.220968_s_at-**NRG1**.206343_s_at	−0.91157	
**C10orf76**.55662_at+NM_018601.220880_at	−0.91218	
**C10orf76**.55662_at+OTUB2.219369_s_at	−0.91235	
**ITGB8**.205816_at+NM_014163.220695_at	−0.91264	
SDC1.201287_s_at+FLJ23172.217016_x_at	−0.91266	
**PRKCB1**.209685_s_at+ACTN1.208636_at	−0.91327	Focal adhesion
BE138647.214314_s_at+AL049242.216101_at	−0.91548	
**TTN**.208195_at/**NEFL**.221805_at	−0.91592	
**KLHL20**.204177_s_at+C19orf50.200076_s_at	−0.91666	
ZNF34.219801_at/FXYD6.217897_at	−0.9181	
**AL359052**.214927_at/**ATP5C1**.205711_x_at	−0.9188	
**KLHL20**.204177_s_at+SDC1.201287_s_at	−0.9188	

We have selected 50 metafeatures (25 most positively correlated and 25 most negatively correlated) and ordered them by Spearman’s rank correlation with MMSE score. Genes in boldface indicate that they were previously discussed in [5] and genes with underlined boldface represent the cases for which the gene has been discussed in the context of AD in the published literature (see File S3 for details).

#### Metafeatures correlating with NFT count

From the list of 50 metafeatures most strongly correlated with *NFT count*, four comprised probe sets targeting genes in common pathways (Table 9). There were also probe sets targeting genes previously investigated in the context of AD (*PPIA*, *TTN*, *MCL1* [89], *UPF1* [90], *RGS4* [91], [92], [93], [94]) and some other genes highlighted by the analysis in Gomez Ravetti et al. [5] (*GNA14*, *C10orf76*, *MMP11*, *TCF7L2*, *COX6B1*, *PRKCI*, *ICA1*).

**Table 9:** Ratio-sum-difference-product metafeatures clustered with NFT count.

Metafeature (Gene Symbol. Probe Set ID)	Correlation Coefficient	Common KEGG Pathways
CCDC121.220321_s_at+U62966.207560_at	0.939664	
CGA.204637_at+PRL.205445_at	0.918887	
AL049435.213817_at+U62966.207560_at	0.912476	
AL049242.216101_at+U62966.207560_at	0.912476	
RNF121.219021_at+**GNA14**.220108_at	0.910216	
U62966.207560_at+WWP1.212637_s_at	0.90442	
MED13L.212207_at+U62966.207560_at	0.897981	
F9.207218_at+U62966.207560_at	0.897533	
U62966.207560_at+**C10orf76**.55662_at	0.890537	
U62966.207560_at+U66059.216597_at	0.884725	
U62966.207560_at+OTUB2.219369_s_at	0.884725	
**MMP11**.203877_at+PRL.205445_at	0.884093	
U62966.207560_at+TEAD3.209454_s_at	0.880455	
U62966.207560_at+SPAG1.210117_at	0.871197	
TEAD1.214600_at+TOX3.216623_x_at	0.867647	
**TTN**.208195_at/CAST.212586_at	0.867043	Focal adhesion
**TTN.**208195_at*LOC100131599.213222_at	0.866901	
**TTN**.208195_at*AP3S1.202442_at	0.866901	
CYP3A7.211843_x_at+PRL.205445_at	0.865975	
U62966.207560_at+RNMT.202684_s_at	0.865512	
**TCF7L2**.212761_at*GNA14.220108_at	0.864466	
LOC286434.222196_at+U62966.207560_at	0.86248	
LOC286434.222196_at+PRL.205445_at	0.861916	
**MCL1.**214057_at+U62966.207560_at	0.861243	
**ITGB8**.205816_at+CAST.212586_at	0.86034	Focal adhesion
EDC4.202496_at+**COX6B1**.201441_at	−0.85128	
CAST.212586_at+**COX6B1**.201441_at	−0.8518	
TMPRSS5.221032_s_at+**COX6B1**.201441_at	−0.85275	
SIRT3.221562_s_at+**COX6B1**.201441_at	−0.8535	
**UPF1**.211168_s_at+**COX6B1**.201441_at	−0.85405	
**COX6B1**.201441_at+ACCN1.206690_at	−0.85484	
**COX6B1**.201441_at+ACTN1.211160_x_at	−0.85501	
NUP98.203195_s_at+**COX6B1**.201441_at	−0.85527	
DET1.219641_at+**COX6B1**.201441_at	−0.85622	
ANKRD34C.216073_at+**COX6B**1.201441_at	−0.85845	
C20orf111.209020_at+**COX6B1**.201441_at	−0.86017	
**PRKCI**.213518_at-HBG2.213515_x_at	−0.86253	
CYP3A7.211843_x_at+CYP26B1.219825_at	−0.86253	Fatty acid metabolism
**COX6B1**.201441_at+DBNDD1.222234_s_at	−0.86441	
UBE3B.213822_s_at+**COX6B1**.201441_at	−0.86685	
IRF2BP1.213771_at+**COX6B1**.201441_at	−0.86907	
**COX6B1**.201441_at+PCSK1.205825_at	−0.87192	
IRF2BP1.213771_at+NM_005758.206809_s_at	−0.87336	
B3GALT2.210121_at+**COX6B1**.201441_at	−0.87405	
SNCG.209877_at+**COX6B1**.201441_at	−0.8754	
**COX6B1**.201441_at/**ATP5C1**.213366_x_at	−0.87976	Oxidative phosphorylation
**COX6B1**.201441_at+**RGS**4.204339_s_at	−0.88473	
ALDOB.217238_s_at-PRL.205445_at	−0.89422	
**ICA1**.207949_s_at+NPAL3.210267_at	−0.89588	
**COX6B1**.201441_at+SORBS2.204288_s_at	−0.89721	

We have selected 50 metafeatures (25 most positively correlated and 25 most negatively correlated) and ordered them by Spearman’s rank correlation with NFT count. Genes in boldface indicate that they were previously discussed in [5] and genes with underlined boldface represent the cases for which the gene has been discussed in the context of AD in the published literature (see File S3 for details).

#### Metafeatures correlating with braak staging

From the list of 50 metafeatures most strongly correlated with *Braak staging*, five comprised probe sets targeting genes in common pathways (See Table 10). Only one gene previously proposed to be involved in AD, *CR1* (see above), was targeted by a probe set within the 50 metafeatures. Probe sets targeting some important genes highlighted by the analysis of Gomez Ravetti et al. [5] (*COX4I1*, *CASP9*, *ITGB1*, *RHOQ*, *DLGAP2*, *GSTA3*, *BCL2*, *COX6A1* and *ATP5C1*) were also found in this set of metafeatures.

**Table 10:** Ratio-sum-difference-product metafeatures clustered with Braak staging.

Metafeature (Gene Symbol. Probe Set ID)	Correlation Coefficient	Common KEGG Pathways
ST3GAL4.203759_at+LRRC48.208140_s_at	0.913812	
LRRC48.208140_s_at*ATP5E.217801_at	0.891149	
LRRC48.208140_s_at*COX4I1.200086_s_at	0.891149	
LRRC48.208140_s_at*DOCK4.205003_at	0.891149	
LRRC48.208140_s_at/SLC6A1.205152_at	0.885391	
PRDM2.216445_at-**CASP9**.210775_x_at	0.88091	
IL12B.207901_at/TNFRSF9.207536_s_at	0.878755	Cytokine-cytokine receptor interaction
C1orf89.220963_s_at+LRRC48.208140_s_at	0.876133	
COPZ2.219561_at-RANBP9.202583_s_at	0.87554	
LRRC48.208140_s_at/CHRDL1.209763_at	0.875476	
LRRC48.208140_s_at-**ITGB1**.216190_x_at	0.874692	
AL110206.216465_at+LRRC48.208140_s_at	0.873511	
AJ251844.216362_at+LRRC48.208140_s_at	0.873511	
SLC47A1.219525_at+PTBP1.211270_x_at	0.871548	
ZNF506.221626_at+LRRC48.208140_s_at	0.871417	
**ITGB1**.216178_x_at+LRRC48.208140_s_at	0.870888	
WWTR1.202133_at*TBC1D5.201813_s_at	0.870138	
MRPS11.215919_s_at+LRRC48.208140_s_at	0.869579	
GJC2.214302_x_at-**CASP9**.210775_x_at	0.868789	
LRRC48.208140_s_at/PTN.209466_x_at	0.868268	
LRRC48.208140_s_at-BCAP29.205084_at	0.867814	
**RHOQ**.212122_at+COPZ2.219561_at	0.866957	
LRRC48.208140_s_at-SLC1A4.209611_s_at	0.865434	
DNAI2.220636_at+ZNF506.221626_at	0.864854	
GJA5.214466_at+LRRC48.208140_s_at	0.864296	
**CR1**.206244_at/SERPINA5.209443_at	−0.66797	Complement and coagulation cascades
**ITGB5**.201125_s_at/TNXB.216333_x_at	−0.86804	ECM-receptor interaction
**CASP9**.210775_x_at-PRDM12.220894_x_at	−0.87283	
**CASP9**.210775_x_at-NTRK3.217033_x_at	−0.87297	
CGGBP1.214050_at-LRRC48.208140_s_at	−0.87301	
**CASP9**.210775_x_at-RENBP.206617_s_at	−0.87347	
KIAA1659.215674_at-LRRC48.208140_s_at	−0.87353	
**CASP9**.210775_x_at-MEOX2.206202_at	−0.87395	
**CASP9**.210775_x_at-**DLGAP2**.216916_s_at	−0.87605	
**CASP9**.210775_x_at-AW408767.217608_at	−0.87684	
**CASP9.**210775_x_at-TRBV6-4.216578_at	−0.87744	
R71245.217654_at-LRRC48.208140_s_at	−0.87744	
LCE2B.207710_at-LRRC48.208140_s_at	−0.8788	
**CASP9**.210775_x_at-FETUB.214417_s_at	−0.87924	
**CASP9**.210775_x_at+**GSTA3**.222102_at	−0.87933	
**CASP9**.210775_x_at*BCL2.203685_at	−0.8806	Apoptosis
**CASP9**.210775_x_at+C6orf106.217924_at	−0.88245	
**ATP5C1**.205711_x_at*COX6A1.200925_at	−0.88297	Oxidative phosphorylation
FLJ22222.219254_at-LRRC48.208140_s_at	−0.88461	
**CASP9**.210775_x_at-X97875.217158_at	−0.88671	
**CASP9**.210775_x_at-ELF3.201510_at	−0.88842	
C15orf39.204494_s_at/LRRC48.208140_s_at	−0.89186	
**CASP9**.210775_x_at-LRRC48.208140_s_at	−0.89462	
**CASP9**.210775_x_at+DENND4A.214787_at	−0.89462	
**CASP9**.210775_x_at-RGS3.220300_at	−0.90202	

We have selected 50 metafeatures (25 most positively correlated and 25 most negatively correlated) and ordered them by Spearman’s rank correlation with Braak staging. Genes in boldface indicate that they were previously discussed in [5] and genes with underlined boldface represent the cases for which the gene has been discussed in the context of AD in the published literature (see File S3 for details).

#### Metafeatures correlating with JSD_control_

From the list of 50 metafeatures most strongly correlated with *JSD_control_*, four comprised probe sets targeting genes in common pathways (Table 11). We also identified metafeatures comprising probe sets targeting various genes highlighted in the analysis in Gomez Ravetti et al. [5] (*RBM19*, *KCNK5*, *AGTR1*, *TUBD1*, *GABRQ*, *MMP11*, *ZNF669*, *TBXA2R*, *NUFIP1*, *LDHA*, *ICA1*).

**Table 11:** Ratio-sum-difference-product metafeatures clustered with *JSD_control_*.

Metafeature (Gene Symbol. Probe Set ID)	Correlation Coefficient	Common KEGG Pathways
**RBM19**.205115_s_at-**KCNK5**.219615_s_at	0.886487	
KLK3.204582_s_at-LOC645961.215320_at	0.875436	
N25732.204131_s_at-MAST3.213045_at	0.85816	
KLK3.204582_s_at-KYNU.210662_at	0.856764	
**RBM19**.205115_s_at-**AGTR1**.208016_s_at	0.856409	
**ZNF669**.220215_at-GEMIN6.219539_at	0.853995	
KLK3.204582_s_at-BG482805.214777_at	0.853527	
KLK3.204582_s_at/MAST3.213045_at	0.852532	
**TUBD1**.210389_x_at-ESR1.205225_at	0.852098	
CASP4.213596_at-LOC90379.221851_at	0.848467	
KLK3.204582_s_at-MLLT4.208512_s_at	0.846819	
**GABRQ**.220886_at+RBMS1.203748_x_at	0.844336	
**MMP11**.203876_s_at-SCGB1D2.206799_at	0.838775	
CASP4.213596_at-AF043586.216394_x_at	0.835322	
**RBM19.**205115_s_at-AF043586.216394_x_at	0.834155	
TMBIM1.217730_at*RBM4.200997_at	0.833134	
AL080106.216121_at-BTN2A2.205298_s_at	0.833121	
**ZNF669.**220215_at-**TBXA2R**.207554_x_at	0.832963	
**ZNF669**.220215_at-LOC90379.221851_at	0.832263	
SLC11A1.217507_at-SCGB1D2.206799_at	0.83103	
KLK3.204582_s_at-CSH2.208342_x_at	0.824365	
CENPE.205046_at-KYNU.210662_at	0.823506	
**ZNF669**.220215_at-SCGB1D2.206799_at	0.823429	
**TUBD1**.210389_x_at-IGSF6.206420_at	0.819997	
U62966.207560_at-**NUFIP1**.205136_s_at	0.817348	
MTHFD1.202309_at/MDH2.213333_at	−0.67409	Glyoxylate and dicarboxylate metabolism
**LDHA**.200650_s_at/GOT2.200708_at	−0.72409	Cysteine metabolism
IRF2BP1.213771_at-AU155105.214782_at	−0.81881	
LUZP4.220665_at-**ZNF669**.220215_at	−0.81961	
TXNDC9.203008_x_at+MAST3.213045_at	−0.82019	
**SMAD3**.205398_s_at-MLLT4.208512_s_at	−0.82047	Adherens junction
ALDOB.217238_s_at+PDE4D.211840_s_at	−0.82091	
BF691447.221484_at*UBP1.218082_s_at	−0.82124	
BF691447.221484_at*RNMT.202683_s_at	−0.82124	
SLC9A3R2.215735_s_at-PSME3.209853_s_at	−0.82473	
LOC90379.221851_at-U66059.216597_at	−0.82552	
TREX1.34689_at-MARCH3.213256_at	−0.82615	
AW408767.217608_at-**MMP11**.203876_s_at	−0.82763	
PURA.213806_at-CENPE.205046_at	−0.8307	
C20orf111.209020_at-PSME3.209853_s_at	−0.83454	
FLJ39739.217136_at-CASP4.213596_at	−0.83709	
ALDOB.217238_s_at-TNFSF14.207907_at	−0.84013	
DIABLO.219350_s_at+MAST3.213045_at	−0.84039	
ALDOB.217238_s_at-CASP4.213596_at	−0.84169	
**ICA1**.207949_s_at+GCNT3.219508_at	−0.84188	
S80491.216974_at-**MMP11**.203876_s_at	−0.84634	
ALDOB.217238_s_at+**SMAD3**.205398_s_at	−0.8545	
AKR1B1.201272_at/KLK3.204582_s_at	−0.86002	
KCNJ5.208397_x_at-CASP4.213596_at	−0.86111	
NDUFA10.217860_at/**ATP5C1**.205711_x_at	−0.86735	Oxidative phosphorylation

We have selected 50 metafeatures (25 most positively correlated and 25 most negatively correlated) and ordered them by Spearman’s rank correlation with *JSD_control_*. Genes in boldface indicate that they were previously discussed in [5] and genes with underlined boldface represent the cases for which the gene has been discussed in the context of AD in the published literature (see File S3 for details).

#### Metafeatures correlating with JSD_severe_

From the list of 50 metafeatures most strongly correlated with *JSD_severe_*, six comprised probe sets targeting genes in common pathways (Table 12). We also identified metafeatures comprising probe sets targeting genes previously studied in the context of AD (*VSNL1*, *PPP2CA*, *CYP3A4* (see above)) and genes highlighted by the analysis in Gomez Ravetti et al. [5] (*PTEN*, *MAPK1*, *COX6A1*, *GABRQ*, *FCAR*, *FZD5*, *PIP5K1C*, *SHANK2*, *CPT2*).

**Table 12:** Ratio-sum-difference-product metafeatures clustered with *JSD_severe_*.

Metafeature (Gene Symbol. Probe Set ID)	Correlation Coefficient	Common KEGG Pathways
TREX1.34689_at+NM_005758.206809_s_at	0.933423	
ESR1.205225_at-AL050026.216626_at	0.910083	
**VSNL1**.203798_s_at-AKAP12.210517_s_at	0.877939	
PURA.213806_at-APOH.205216_s_at	0.872983	
**PTEN**.222176_at-AL050026.216626_at	0.870794	
**MAPK1**.208351_s_at+MLLT4.208512_s_at	0.867901	Adherens junction
TREX1.34689_at*COX6A1.200925_at	0.864481	
CNOT1.200861_at-**GABRQ**.220886_at	0.856692	
UBE3B.213822_s_at+ADK.204119_s_at	0.847332	
MTSS1.210360_s_at-**PTEN**.211711_s_at	0.843329	
S80491.216974_at-**PTEN**.211711_s_at	0.842751	
MLLT4.208512_s_at/**PTEN**.211711_s_at	0.842239	Tight junction
**PPP2CA**.208652_at/**PTEN**.211711_s_at	0.842212	Tight junction
P2RY10.214615_at-AL050026.216626_at	0.842191	
**TTK**.204822_at-**PTEN**.211711_s_at	0.840396	Inositol phosphate metabolism
FLJ39739.217136_at-**PTEN**.211711_s_at	0.837966	
UBE3B.213822_s_at+ATRN.212517_at	0.836922	
TREX1.34689_at+RPS24P2.217188_s_at	0.83673	
UBE3B.213822_s_at-FOLH1.217483_at	0.835834	
SCGB2A1.205979_at-APOH.205216_s_at	0.83274	
AJ302559.216818_s_at-KLK3.204582_s_at	0.830253	
TREX1.34689_at-PRKD2.38269_at	0.82812	
AJ302559.216818_s_at-**PTEN**.211711_s_at	0.827864	
TPD52.201691_s_at-FOLH1.217483_at	0.827066	
TREX1.34689_at+DDX18.208897_s_at	0.8255	
SI.206664_at+**PTEN**.211711_s_at	−0.80904	
TNRC4.215045_at*AL536319.212606_at	−0.80962	
**FCAR**.211307_s_at+**PTEN**.211711_s_at	−0.81072	
AL050026.216626_at+OTUB2.219369_s_at	−0.81137	
CMKLR1.210659_at+**PTEN**.211711_s_at	−0.81349	
KLK3.204582_s_at+**PTEN**.211711_s_at	−0.81565	
PAX3.216059_at+NM_018601.220880_at	−0.82244	
AL050026.216626_at+CTAGE5.204055_s_at	−0.82288	
**FZD5**.206136_at+**PTEN**.211711_s_at	−0.82362	
CAMP.210244_at-SCGB1D2.206799_at	−0.82501	
**PTEN**.211711_s_at*PIP5K1C.212518_at	−0.82503	Phosphatidylinositol signaling system
PLA2G2F.215870_s_at-RARRES2.209496_at	−0.82717	
BPI.205557_at+**PTEN**.211711_s_at	−0.8328	
AI478300.217526_at-TREX1.34689_at	−0.83316	
AL050026.216626_at+**PTEN**.211711_s_at	−0.83401	
**PTEN**.211711_s_at-PTPRN2.203030_s_at	−0.83583	
FOLH1.217483_at-SCGB1D2.206799_at	−0.83998	
RAB14.200928_s_at+**PTEN.**211711_s_at	−0.84169	
GALNT10.212256_at*SHANK2.213307_at	−0.84811	
NM_004908.208254_at+**PTEN**.211711_s_at	−0.84856	
SPRED2.212458_at*DDN.214788_x_at	−0.85116	
FADS1.217462_at+**PTEN**.211711_s_at	−0.86774	
**CYP3A4**.205998_x_a/**CPT2**.204264_at	−0.87018	Fatty acid metabolism
N25732.204131_s_at/AF043586.216394_x_at	−0.9031	
C3orf63.209285_s_at*DDN.214788_x_at	−0.90361	

We have selected 50 metafeatures (25 most positively correlated and 25 most negatively correlated) and ordered them by Spearman’s rank correlation with *JSD_severe_*. Genes in boldface indicate that they were previously discussed in [5] and genes with underlined boldface represent the cases for which the gene has been discussed in the context of AD in the published literature (see File S3 for details).

### Comparison of Metafeature Correlations and Single Probe Set Correlations

The observation of particular probe sets recurring in multiple clustered metafeatures raises the question of whether the clustering of certain metafeatures is driven by a strong correlation between a progression marker and only one of the two individual probe sets comprising a metafeature. To investigate this possibility, we separately assessed the correlation of the two probe sets comprising a metafeature with the progression marker in question and compared this to the correlation between the metafeature and the progression marker.

Deeper analysis of the “ratio metafeatures” that clustered with MMSE scores reveals a number of metafeatures where individual probe sets (one or both) are not significantly correlated (*p*>0.05) with MMSE (see Table S1). This suggests that the high correlation of certain metafeatures does not simply reflect an additive effect of its two component probe sets (see Figure S6) but instead is driven by the dynamics of the interrelationship (possibly a biological interaction) between the two transcripts that are targeted.

**Figure 2:**
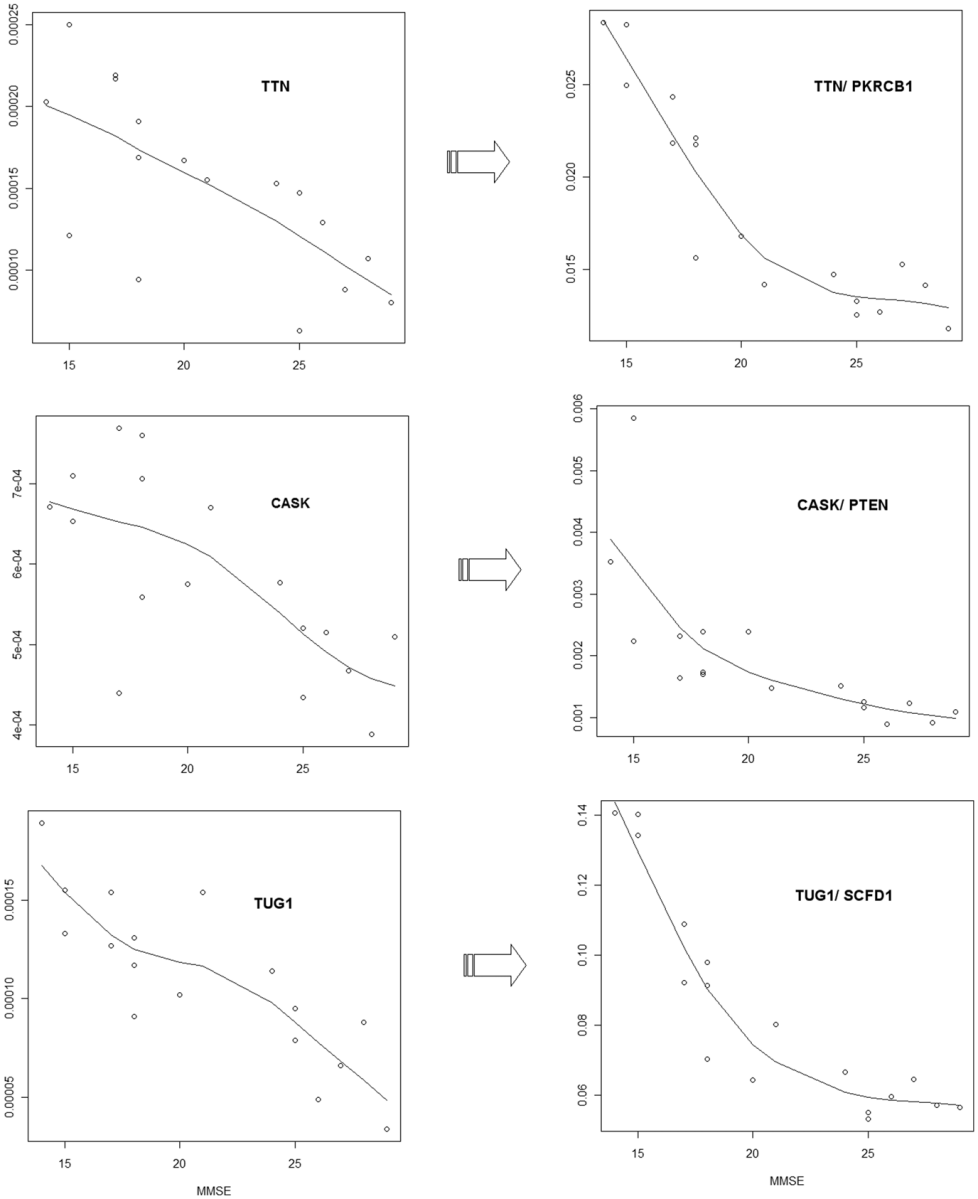
Comparison of single probe set correlations and metafeature correlations. Figure shows plots of the correlation with *MMSE score* of three probe sets targeting *TTN*, *CASK* and *TUG1* and three metafeatures involving these probe sets (*TTN*/*PKRCB1*, *CASK*/*PTEN* and *TUG1*/*SCFD1*). In this example, the correlations between MMSE score and the metafeatures are much better than the correlation between MMSE score and the individual probe sets.

For specific examples we refer to Figure 2 where we demonstrate an example scenario with the three probe sets targeting *TTN*, *CASK* and *TUG1* and the metafeatures *TTN*/*PKRCB1*, *CASK*/*PTEN* and *TUG1*/*SCFD1*. In this example, the metafeatures show a better correlation with MMSE score than the relevant individual probe sets (Figure 2). In general, we found that if both the probe sets in a metafeature target genes in a common pathway, then the metafeature shows better correlation with the progression marker than either of the two individual probe sets. For example, *TTN* and *PKRCB1* both appear in the ‘focal adhesion’ KEGG pathway and *CASK* and *PTEN* in both appear in ‘tight junction’ pathway.

There were various instances in which the individual components of highly correlated metafeatures did not map to a common KEGG pathway. For example, a metafeature containing probe sets for *TUG1* and *SCFD1* shows a better correlation with MMSE score than either probe set individually. However it is not surprising that these transcripts do not map to a common pathway, as long ncRNAs such as TUG1 are relative newcomers to functional annotation.

It should be noted, however, that a high proportion of the metafeatures (>90%) showed comparatively better correlations with the progression markers than the individual probe sets comprising these metafeatures. While this is not a universal phenomenon (see Figure S6 and Table S1 for examples, where two probe sets that are highly correlated with a progression marker combine to create a poorly correlated metafeature), in view of the larger data space occupied by the metafeatures, it is logical that the metafeature analysis may yield an increased proportion of spurious, false positive results. This is supported by the observed difference in the estimated false discovery rate of two datasets of different sizes (see Table S2 and Table S3). In an attempt to avoid such results, we subsequently focus on ‘robust’ findings – probe sets that recurrently cluster with different markers of AD progression.

### Robust Markers of AD Progression

**Figure 3:**
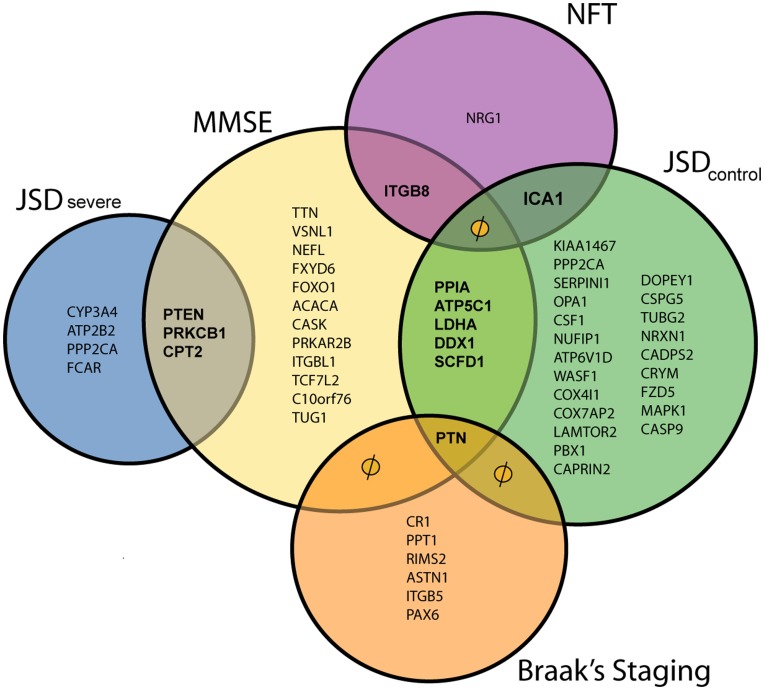
Venn diagram of the different transcripts clustered with progression markers in the 941,885 metafeatures data set. This figure highlights the ‘robust correlating’ transcripts that are shared by different progression marker clusters. A null (φ) symbol here means that even if an overlap is shown in the figure, there is no common transcript. We refer the readers to Supporting Information Table S4., for further details of correlation of these markers to the phenotypes.

**Figure 4:**
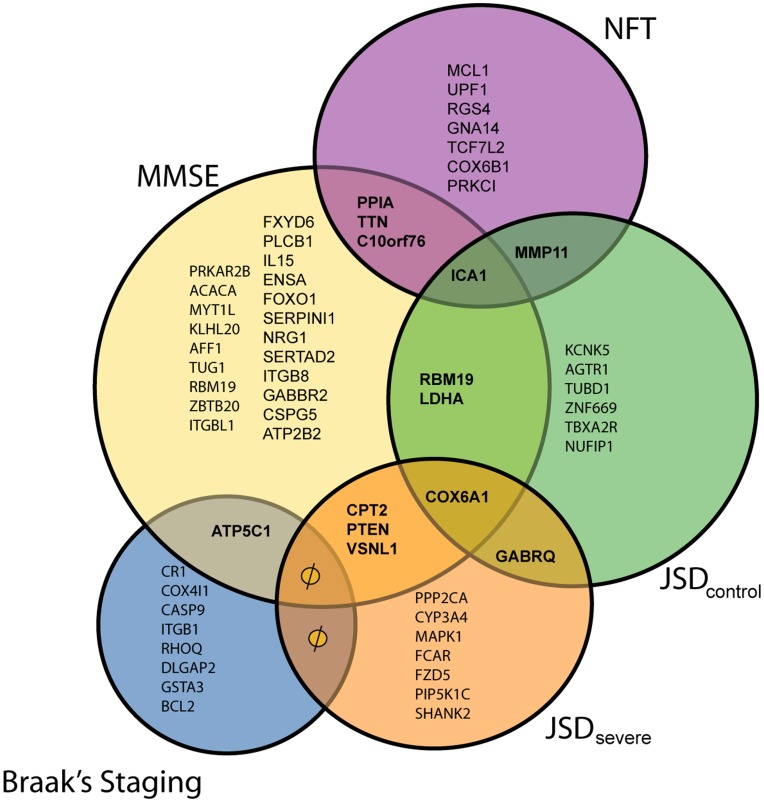
Venn diagram of the different transcripts clustered with progression markers in the 3,763,403 metafeatures data set. This figure highlights the ‘robust correlating’ transcripts that are shared by different progression marker clusters. A null (φ) symbol here means that even if an overlap is shown in the figure, there is no common transcript. We refer the readers to Supporting Information Table S5., for further details of correlation of these markers to the phenotypes.

We next attempted to identify probe sets that appeared recurrently in the metafeatures and also clustered with different markers of AD progression. We depict this group of probe sets in a 5-way Venn diagram (Figure 3 and Figure 4). In these figures, a null (φ) symbol means that even if an overlap is shown in the figure, there is no common transcript. From the 941,885 *ratio* metafeatures data set, we identified 11 probe sets that, as part of metafeatures, clustered with more than one progression marker. The genes targeted by these probe sets were *PPIA*, *ATP5C1*, *LDHA*, *DDX1*, *SCFD1*, *ITGB8*, *PTEN*, *PRKCB1*, *CPT2*, *ICA1* and *PTN*. From the 3,763,403 *ratios-sum-difference-product* metafeatures data set, there were 13 probe sets that, as part of metafeatures, clustered with more than one progression marker. The genes targeted by these probe sets were *PPIA*, *TTN*, *C10orf76*, *ICA1*, *MMP11*, *RBM19*, *LDHA*, *COX6A1*, *GABRQ*, *CPT2*, *PTEN*, *VSNL1* and *ATP5C1*. Notably, six genes were identified as clustering with more than one progression marker in both metafeature datasets: *PPIA*, *ATP5C1*, *LDHA*, *PTEN*, *CPT2* and *ICA1*. We refer the readers to the Table S4 and Table S5, for further details of correlation of these markers to the phenotypes.

### Validation of Robust Markers in an Alternative Dataset

**Figure 5:**
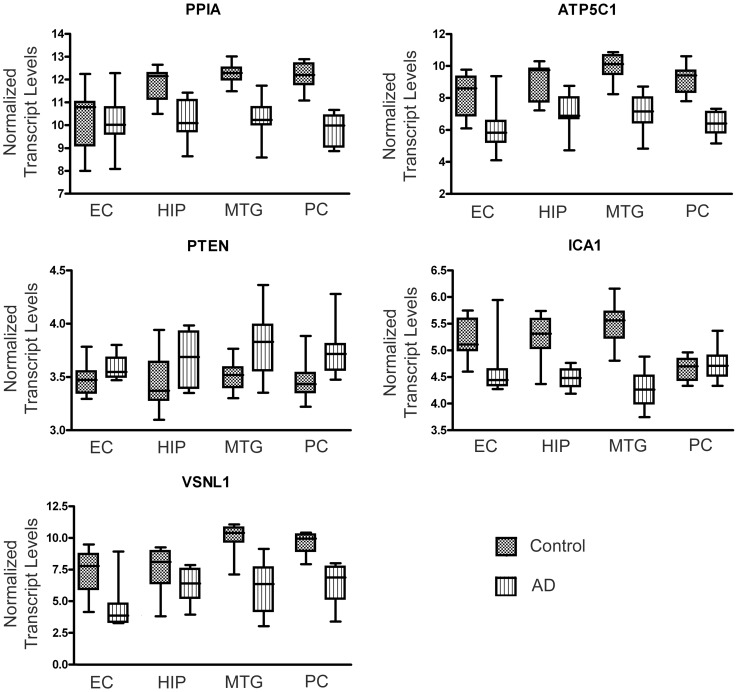
Validation of robust markers of AD progression in an alternative dataset. Transcript levels for selected genes of interest were investigated in the microarray dataset of Liang and colleagues [95], [96], which assessed gene expression in healthy neurons isolated from four different regions of control and AD brain: entorhinal cortex (EC), hippocampus (HIP), middle temporal gyrus (MTG) and posterior cingulate cortex (PC). Data presented in this figure were normalized using Robust Multichip Average (RMA). In the box and whisker plots, the bottom and top of the box represent the lower and upper quartiles, respectively, and the band within the boxes represents the median, while the ends of the whiskers represent the minimum and maximum values.

In order to gain insights into whether the robust markers highlighted above are restricted to the hippocampus or show changes in other AD-affected brain regions, we utilized an independent dataset contributed by Liang and colleagues [95], [96]. This dataset contains microarray data on gene expression in neurons isolated from four different regions of control and AD brain: entorhinal cortex (EC), hippocampus (HIP), middle temporal gyrus (MTG) and posterior cingulate cortex (PC). Molecular signatures of each different region were generated as described in Materials and Methods. Several of the ‘robust’ probe set markers highlighted by our current analysis of hippocampal tissue were also selected in the molecular signatures of two or more regional neuronal populations. For example, *PPIA* and *ATP5C1* showed expression changes in neurons isolated from the MTG and PC of AD brain relative to control brain, *PTEN* showed expression changes in the HIP and PC and *ICA1* showed expression changes in the HIP, MTG and PC (Figure 5). In addition, Visinin-like 1 (*VSNL1*), highlighted in the analysis in Gomez Ravetti et al. [5] as one of the best markers of AD progression and recently proposed as one of the four best CSF biomarkers of early AD [97], showed expression changes in neurons isolated from EC, MTG and PC (Figure 5) and has been shown in an additional dataset to have altered expression in various brain regions in AD [98].

## Discussion

The present study has extended on our previous analysis in [5] by (i) considering variables that represent the interrelationship between two RNA transcripts (i.e. metafeatures) and (ii) applying a novel and powerful graph-based clustering approach to identify a reduced set of transcripts or transcript pairs that correlate strongly with markers of AD progression. This clustering approach is facilitated by the implementation of an external memory algorithm on a graphical processing unit, which allows clustering of massive datasets (in this case involving four million elements) that is not feasible using standard computational methods.

In addition to identifying transcripts and metafeatures that correlate with established phenotypic markers of AD severity (i.e. MMSE score, NFT count, Braak staging), we utilized two addition quantifiers of AD progression, based on Jensen-Shannon divergence, to identify transcripts and metafeatures that correlate with a putative molecular trend from control to severe AD. It remains to be seen whether these quantifiers provide a more accurate assessment of AD severity, however there are several promising attributes that suggest this is the case. Firstly, the Jensen-Shannon divergence values are based on a set of 1,372 different transcript markers, in contrast to the univariate markers of neuropathology or cognitive function. Secondly, some of the changes observed at the transcriptional level may underlie AD pathogenesis, whereas phenotypic consequences are more likely to simply reflect the molecular perturbations that drive disease pathogenesis. The results presented here indicate that a number of probe sets that are highly correlated with Jensen-Shannon divergence are also highly correlated with more traditional phenotypic markers of AD progression. Our previous studies using these quantifiers in the context of AD [5] and cancer [99] have also yielded high consensus with established markers of disease progression. Together, these findings give us confidence that metrics based on multivariate transcriptional changes can act as reliable markers of disease stage.

The analyses reported here demonstrate that, although there exists a relatively large molecular signature associated with AD progression, a relatively small number of transcripts appear recurrently in metafeatures clustered with the progression markers. This allowed a focussed investigation of a reduced set of biomarkers that have been previously studied in the context of cognitive decline and AD. Furthermore, our approach also put emphasis on a few novel markers that have not been discussed previously in relation to AD progression and warrant further investigation.

While it is outside the scope of this paper to discuss in detail all of the genes identified in the analyses, focussed discussion of some of the most robust findings is warranted. As mentioned at the end of the Results, as set of six genes (*PPIA*, *ATP5C1*, *LDHA*, *PTEN*, *ICA1*, *CPT2*) recurrently appeared in metafeatures and clustered with more than one progression marker in both metafeature datasets. Furthermore, changes in expression of several of these genes were validated in an alternative microarray dataset of neurons from different AD-affected brain regions, lending further support to the proposal that altered expression of these genes may be involved in AD pathogenesis.

Peptidylprolyl isomerase A (PPIA), also known as cyclophilin A, is believed to accelerate protein folding. Evidence from neural cell lines suggests PPIA can protect against Aβ-induced oxidative stress, possibly by acting as a ROS scavenger [100]. Proteomics studies have revealed decreased expression of PPIA in brains of patients with non-Alzheimer’s disease tauopathies [41], suggesting alterations in PPIA may be associated with the general process of neurodegeneration rather than AD specifically. Curiously, *PPIA* has been proposed as a suitable reference gene for PCR studies of AD brain due to its stable expression [101], [102]. The results of our analysis strongly argue against this and instead indicate that *PPIA* expression, particularly when considered as part of a metafeature, is strongly correlated with AD progression.

ATP synthase subunit gamma (ATP5C1) encodes a subunit of mitochondrial ATP synthase, important for catalyzing ATP synthesis in oxidative phosphorylation. While this gene has not previously been implicated in AD, its transcriptional correlation with AD progression may reflect disturbances in energy production as a result of cellular loss.

Lactate dehydrogenase A (LDHA), another metabolic gene, is responsible for catalysing the conversion of lactate to pyruvate, the final step in anaerobic glycolysis. A recent study has demonstrated that increased LDHA activity is a feature of nerve cell lines that are resistant to Aβ-induced cell death and that the phenomenon of aerobic glycolysis might contribute to the mechanisms by which certain neurons in the AD brain survive apoptosis [103].

Phosphatase and tensin homolog (PTEN) has generally been studied in the context of cancer, as it is a tumor suppressor with phosphatase activity that negatively regulates the AKT/PKB signalling pathway. However PTEN has also been shown to be necessary for proper migration of neurons and glia [104]. There is decreased expression and altered distribution of PTEN in AD brain [105], [106], where it localizes with neuritic pathology such as neurofibrillary tangles in damaged neurons [36]. PTEN affects phosphorylation and aggregation of tau [106], [107] and appears to be regulated by presenilin, as presenilin deficient neurons show a substantial reduction in PTEN [108]. Furthermore, mutations in the PTEN induced putative kinase 1 (PINK1) gene have been linked to early-onset familial Parkinson’s disease [109], [110], while ablation of PTEN in dopaminergic neurons is neuroprotective in mouse models of Parkinson’s disease [111].

Islet cell autoantigen 1 (ICA1) is believed to be an autoantigen in insulin-dependent diabetes mellitus. ICA1 is the major binding partner of PICK1 and together these proteins regulate trafficking of AMPA glutamate receptors to the synapse [112]. It has also been proposed that ICA1 participates in the process of neuroendocrine secretion through association with certain secretory vesicles [113].

Carnitine palmitoyltransferase 2 (CPT2) is involved in the oxidation of long-chain fatty acids in the mitochondria. This gene has not previously been associated with AD.

In addition, we highlight some relevant genes that correlated with more than one progression marker in one of the metafeature datasets. Protein kinase C beta, encoded by PRKCB1, is involved in a wide range of signalling pathways. Increased expression of protein kinase C beta has been observed in membrane fractions of aged Tg2576 mice, a model of AD [114]. Furthermore, one of the best biomarkers in [5], visinin-like 1 (VSNL1), is only expressed in neurons and shows decreasing expression as AD progresses. Levels of VSNL1 in the CSF have recently been proposed as an effective biomarker of early AD [97].

It is interesting to remark that the individual markers identified in this study are bringing new insights to the pathological mechanisms involved in AD but that an integrative approach is required to understand them. For example, increased VSNL1 in the CSF observed in [97] may be a consequence of increasing neuronal death rather than an marker of early AD. On the other hand, as LDHA expression is currently being considered as a possible marker of aerobic glycolysis in Aβ-resistant neurons [103], the correlation between LDHA expression and AD progression makes sense if we think that Aβ-resistant neurons will be proportionally more abundant in samples with greater disease severity.

One limitation of the present study is that the low number of samples (17) available for these analyses may have resulted in a large number of highly correlated probe sets or metafeatures that are false positives (see Figure S2, Figure S3, Figure S4, Figure S5, for a validation of our correlations). The selection of just a few features out of this large data space has been a critical task that we attempted to solve by focusing on those which appear most recurrently. Unfortunately, there are strong possibilities that even with a set of random data and a very large search space, a set of false positive markers may recurrently appear. However, since a relatively higher number of published AD studies can already be found that implicate these markers, we feel comfortable in making the claim that they warrant further investigation in future AD research.

The results presented here support the hypothesis that systematically considering relationships between two or more features (“metafeatures”) can improve biomarker discovery, particularly when explored within a multivariate framework. While univariate approaches may still provide important and complementary insights to those obtained using multivariate methods, we believe that utilizing both approaches in conjunction is likely to produce the most robust and relevant findings. Computational advances such as the external memory implementation of our clustering algorithm will hopefully make investigations of this type more commonplace, and we are currently working towards more sophisticated parallel applications that would enable the study even of larger datasets across a range of diseases.

## Materials and Methods

### Datasets

This analysis draws on the data set contributed by Blalock et al. [3] which can be accessed from the NCBI Gene Expression Omnibus under the accession number GSE1297. The Blalock study used Affymetrix HG-U133A microarrays to generate data on 22,286 probe sets. From this dataset, we focused our analysis on the 1,372-probe set signature identified by Gomez Ravetti et al. [5].

The Blalock study assessed gene expression in hippocampal tissue samples from 31 participants. Participants were categorized into one of four clinical groups using the MMSE criterion: “Control” (MMSE >25, *n* = 9),“Incipient AD” (MMSE 20–26, *n* = 7), “Moderate AD” (MMSE 14–19, *n* = 8) or “Severe AD” (MMSE <14, *n* = 7). Borderline cases (e.g. MMSE = 26) were resolved using NFT count and Braak staging data [115].

In the present study, instead of using all 31 samples, we excluded 14 samples that had gene expression profiles similar to the representative profile of the “Control” group (*n* = 7) or similar to the representative profile of the “Severe AD” group (*n* = 7). The 17 remaining samples correspond to the central 17 columns of the supplementary material ‘File S2 (sheet:1372-probe)’ of [5] and the data set containing the signature of Gomez Ravetti et al. [5] for these 17 samples is termed *1,372-probe set signature* throughout the paper. Our rationale for excluding these 14 samples is two-fold. Firstly, little information about disease progression is likely to be gained by considering participants at either extreme of a disease spectrum. Control participants will not have developed any molecular characteristics of early AD and participants with severe AD may have already progressed to the disease endpoint. Secondly, as the “Control” and “Severe AD” groups were used to generate the 1,372-probe set signature, inclusion of these samples would likely influence correlations in a biased way. By excluding these samples, we can assess correlation with AD progression in a truly independent ‘test’ set of samples.

The advantages of using pair-wise relational features (i.e. metafeatures) have recently been demonstrated by Rocha de Paula et al. [116] in the context of plasma protein biomarkers for the early detection of AD. We therefore expanded the 1,372-probe set signature by applying different operators between each possible pair of probe sets. This led to the creation of two “artificial” data sets:

- The first data set includes all the pair-wise *ratios* of the gene expression values in 1,372-probe set data. It contains a total of 941,885 probe sets, metafeatures and progression markers. We refer to this data set as the *941,885 ratio metafeatures data set*.
- The second data set includes all the pair-wise *differences*, *summations*, *ratios* and *products* of the gene expression values in 1,372-probe set data. It contains a total of 3,763,403 probe sets, metafeatures and progression markers. We refer to this data set as the *3,763,403 ratio-sum-difference-product metafeatures data set*.

In each of these two data sets, we have included the original 1,372-probes gene expression signature and five measures of AD progression: MMSE score, NFT count and Braak staging from Blalock et al. [3] and the *Jensen-Shannon divergences*, *JSD_control_* and *JSD_severe_*, from Ravetti et al. [5]. We assume here that correlation between the values associated with these measures and microarray probe set expression values can highlight important biomarkers of AD progression.

### Data Clustering

We have adapted, enhanced and re-implemented the MST*k*NN graph partitioning algorithm in [10] to cluster the features in the large data sets. The proposed method utilizes a graph partitioning approach that optimizes both the local minimum (by using the *k*NN graph) and global minimum (by using the MST). Therefore, the clusters presented are not necessarily a sorted a list of probes by their correlation to the phenotypes.

The algorithm first constructs an undirected and complete graph from the data set where each node is a biological feature and each edge represents a correlation between two features. Then, the algorithm starts the clustering process by computing two proximity graphs: a minimum spanning tree (*G_MST_*) and a *k*-nearest neighbour graph (*G_kNN_*); where the value for *k* is adaptively selected from the following equation:
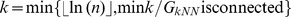
(1)


Subsequently, the algorithm inspects all edges in *G_MST_*. If for a given edge (*x,y*) neither *x* is one of the *k* nearest neighbors of *y*, nor *y* is one of the *k* nearest neighbors of *x*, the edge is eliminated from *G_MST_*. This results in a new graph 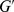 = *G_MST_* – {(*x,y*)}. Since *G_MST_* is a tree, after the first edge is deleted 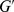 is now a *forest*, as it is a graph that composed of two subtrees. The algorithm continues applying the same procedure to each subtree in 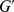 thus generated (with a value of *k* re-adjusted by eq. (1) above where *n* is now the number of nodes in each subtree), until no further partition is possible. The final partition of the nodes of 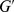 induced by the forest is the result of the clustering algorithm.

**Figure 6:**
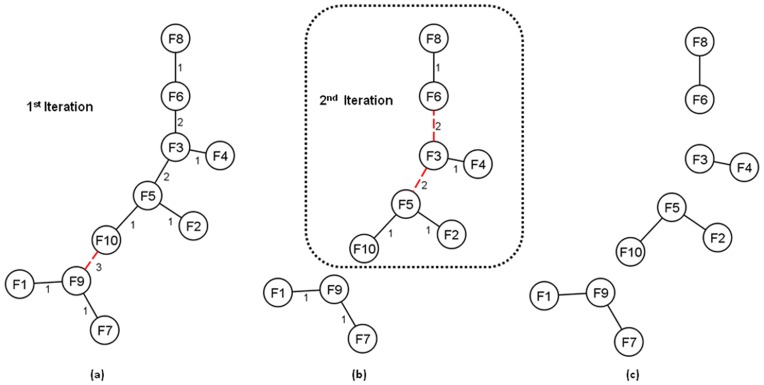
Demonstration of the modified MSTkNN algorithm. (a) An MSTp created from a data set with n=10 features/probe sets. Each edge is labeled with an integer value p, where the value of p is determined using a sorted list of nearest neighbors for each feature (see eq. (2)). The edge between F9 and F10 is a candidate for elimination, since it has a value of p > = 2 (b) Two connected components are identified and we apply the same procedure with the component that has more than three elements. (c) The final outcome of the clustering.

**Figure 6. Demonstration of the modified MST** ***k*** **NN algorithm. (a) An MST** ***p*** **created from a data set with** ***n*** **= 10 features/probe sets.** Each edge is labeled with an integer value *p*, where the value of *p* is determined using a sorted list of nearest neighbors for each feature (see eq. (2)). The edge between F9 and F10 is a candidate for elimination, since it has a value of *p*> 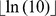 = 2 (b) Two connected components are identified and we apply the same procedure with the component that has more than three elements. (c) The final outcome of the clustering.

The original algorithm requires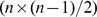 distance values (between all pairs of the *n* elements) as the input. For a large data set, this may be too large to fit in the computer’s in-memory and, for even larger values of *n,* it may not even fit in external memory. Even if we can store the distance matrix in the external memory, the computational speed will slow down dramatically because of the increased number of I/O operations. Therefore, we modified this step and instead of creating the complete graph from the distance matrix, we create a *q*-nearest neighbor graph (*G_qNN_*), where *q* = 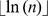
+1. This procedure reduces the input graph size, but still creates a reasonable clustering structure of the data set. The value of the *q* is determined from the *inclusion relationship* [117] of the *G_MST_* and the family of the nested sequence of graphs (*G_kNN_*, where *k* > ln(*n*)).

Next, we compute the MST of the *G_qNN_* graph. We term it as, *G_MSTp_*. We annotate each edge in *G_MSTp_* according to the following procedure: for each edge (*a*,*b*) in *E*(*G_MSTp_*) we assign an integer value *p* such that if *f(a,b)* is the index of *b* in the sorted list of nearest neighbors of *a* in *G_qNN_*, the value of *p* is given by,
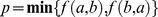
(2)


We define the maximum value of *p* in the MST*p* (or any of its components) as *p_max_* and then, we partition the *G_MSTp_* with the following criteria:

*C*_1_. If *p*> 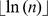; remove the edge,

*C*_2_. If *p_max_* < 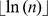; remove the edges with weight *p_max_ –*1, and;

*C*_3_. If *p_max_* = 1 or *p_max_* = 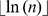; do not remove any edge and the result is a “cluster”.

The final output of our algorithm is a set of partitions or clusters of the input data (See Figure 6). The algorithm does not require any pre-determined value for *q* but it is possible to change the threshold from 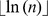 to any other user-defined parameter. The complete algorithm can be found in [13]. To accelerate the data preprocessing we employed General Purpose Graphics Processing Unit (GPGPU) computing and implemented a fast and scalable approach to compute the distance metrics and the *q*-nearest neighbor graph (*G_qNN_*). An illustrated example of our GPU-based nearest neighbor search algorithm is given in File S4.

To create the MST from large data set we adapted the EM MST algorithm in [11] and modified it to annotate the edges according eq. (4). The I/O complexity of this algorithm is O(sort(*m*)•log(*n*/*M*)), where *n* is the number of nodes of the original graph, *m* is number of edges and *M* number of nodes that fit into computer’s internal memory, respectively, and the sort(*m*) is the time required for sorting the *m* edges. After partitioning the MST, we identify the connected components using the EM connected component algorithm in [11], [12], [118]. The I/O complexity of this algorithm is *O*(*m*•log(log(*n*)). Unlike other clustering tools, we store the connected components/clusters in external memory and only keep the *list* of the components in computer’s in-memory. This eliminates the excessive use of the in-memory even when there are a large number of components or clusters. Additionally, we tuned the implementations of the adapted algorithms [11], [12],[118] for better performance with denser graphs. Since our algorithm has been implemented in external memory approach, we term our algorithm as EM MST*k*NN algorithm. Please note here that our proposed method can be implemented either in-memory or external memory paradigm. To make this method further scalable, we have taken the advantages of external memory algorithms and environments.

The computational tests were performed on a Xenon Nitro T5 Supermicro server (16 CPU cores, 32 GB RAM, 4x NVIDIA Tesla C2050 “Fermi” GPU cards (1792 GPU Cores and 12 Gb RAM total) and 800GB Hard-disk) and the programs were written in C/C++ with the support of CUDA [119], STL, STXXL [120] and BOOST [121] library and compiled using the g++ and nvcc compiler on a Linux operating system with kernel version 2.6.9.

### Monte-Carlo Random Permutation Test

Assessing correlations involving a large number of metafeatures and a small number of samples has the potential to lead to spurious, false positive results. To estimate the false discovery rate when assessing correlations involving metafeatures, we performed a simple a Monte-Carlo permutation test, a useful resampling test when there are many possible orderings of the samples. In this test, we randomly permuted (rearranged) the values of the progression marker in question and computed the correlation of each metafeature against it. A total of 1,000 iterations of the test were performed and the average number of metafeatures passing various correlation coefficient thresholds determined.

### Functional Annotation

After performing the clustering on the expanded data sets, we identified the specific clusters that contained the progression markers (MMSE score, NFT count, Braak staging, *JSD_control_* and *JSD_severe_*) and analysed the correlation (using Spearman’s rank computation) of the clustering probe sets or metafeatures with these progression markers. If the size of the cluster was very big, we noted the top most positively and negatively correlated probe sets or metafeatures.

GATHER [122], a popular online tool for interpreting genomic signatures, was used to assess possible biological relationships between the two transcripts targeted by the probe sets comprising a metafeature. We checked each of the clustering metafeatures to determine if the relevant transcripts share any common biological pathway (KEGG pathways). Our objective here is to detect the pair of transcripts that not only appear in the same pathway but also jointly activate the progression of AD.

### Validation Using an Alternative Dataset

For validation of changes in select genes, we analyzed the dataset contributed by Liang and colleagues [95], [96], which can be accessed from NCBI Gene Expression Omnibus under the accession number GSE5281. This microarray dataset was generated using Affymetrix Human Genome U133 Plus 2.0 Arrays and assessed gene expression in healthy neurons isolated by laser capture microdissection from different regions of post-mortem control and AD brain. We refer the reader to [95], [96] for full experimental details.

In the analyses presented here, we investigated gene expression in the entorhinal cortex (13 controls, 10 AD), hippocampus (13 controls, 10 AD), middle temporal gyrus (12 controls, 16 AD) and posterior cingulate cortex (13 controls, 9 AD). Microarray data were normalised with RMA in the Affymetrix Expression Console (v1.1). For each region, genetic signatures that discriminate control and AD samples were generated as described in [5]. Briefly, data were first preprocessed by discretization of the expression values using an implementation of Fayyad and Irani’s algorithm [6], an entropy-based heuristic. This was followed by a filtering step to discard probe sets that do not provide sufficient information to discriminate between the control and AD classes, based on the Minimum Description Length principle (reviewed in [123]). The matrix of discrete values returned after entropy filtering was then used to create an instance of the *(α,β)-k-Feature Set problem* (for details see [123]). The optimal solution to this problem was used as the genetic signature.

## Supporting Information

Figure S1 **Comparison of the clustering outcomes with stringent** ***p*** **-values.**
(DOC)

Figure S2 **Frequency of the highly negative correlations in the 941,885 ratio metafeatures data set.**
(DOC)

Figure S3 **Frequency of the highly positive correlations in the 941,885 ratio metafeatures data set.**
(DOC)

Figure S4 **Frequency of the highly negative correlations in the 1,372-probe AD signature data set.**
(DOC)

Figure S5 **Frequency of the highly positive correlations in the 1,372-probe AD signature data set.**
(DOC)

Figure S6 **Counter example for the correlation of probe set pairs.**
(DOC)

Table S1 **Analysis of the 941,885 ratio metafeatures clustered with the MMSE score.**
(DOC)

Table S2 **False discovery rate (FDR) in 1,372-probe AD signature data set.**
(DOC)

Table S3 **False discovery rate (FDR) 941,885 ratio metafeatures data set.**
(DOC)

Table S4 **Pair-wise comparisons of overlap between progression marker clustering outcomes in the 941,885 metafeatures data set.**
(DOC)

Table S5 **Pair-wise comparisons of overlap between progression marker clustering outcomes in the 3,763,403 metafeatures data set.**
(DOC)

File S1 **Clusters containing the progression markers (from the 1,372-probe AD signature data set).**
(XLSX)

File S2 **Clusters containing the progression markers (from the 941,885 ratio metafeatures data set).**
(XLSX)

File S3 **Clusters containing the progression markers (from the 3,763,403 ratio-sum-difference-product metafeatures data set).**
(XLSX)

File S4 **Implementation of the proposed clustering method.**
(PDF)
