# Supplementary material for: Unveiling Clusters of RNA Transcript Pairs Associated with Markers of Alzheimer’s Disease Progression
Source: PLoS One. 2012 Sep 21;7(9):e45535. doi: 10.1371/journal.pone.0045535 (PMC3448659; doi:10.1371/journal.pone.0045535)
Supplement: Figure S1 — Comparison of the clustering outcomes with stringent p -values. (DOC) [file pone.0045535.s001.doc]

**Figure S1. Comparison of the clustering outcomes with stringent *p*-values.**


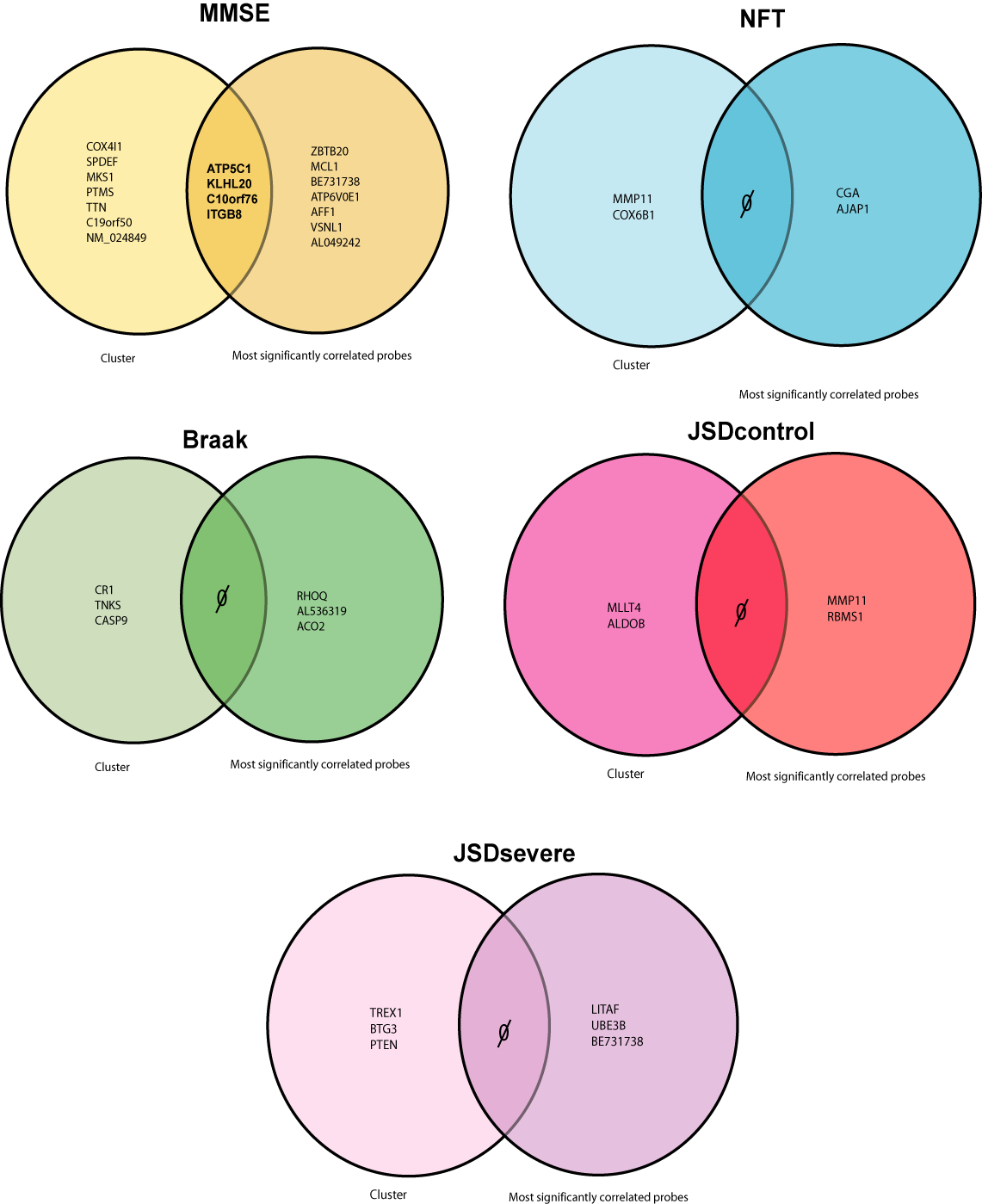


Similarities and differences between the findings of the clustering experiments with findings when considering the same number of most highly correlated probes. For example, we compared the 11 probes the clustered with MMSE to the 11 probes most significantly correlated with MMSE using standard statistical methods. While there were some similarities, there were also differences, and some genes with particular relevance to AD were selected by the clustering method but not by filtering based on statistical significance. For example, clustering with Braak staging identified CR1 which, as mentioned above, is proposed as a genetic risk factor for AD, however this probe was not identified by the statistical method.
