# Supplementary material for: Unveiling Clusters of RNA Transcript Pairs Associated with Markers of Alzheimer’s Disease Progression
Source: PLoS One. 2012 Sep 21;7(9):e45535. doi: 10.1371/journal.pone.0045535 (PMC3448659; doi:10.1371/journal.pone.0045535)
Supplement: Figure S3 — Frequency of the highly positive correlations in the 941,885 ratio metafeatures data set. (DOC) [file pone.0045535.s003.doc]

**Figure S3. Frequency of the highly positive correlations in the 941,885 ratio metafeatures data set.**

**
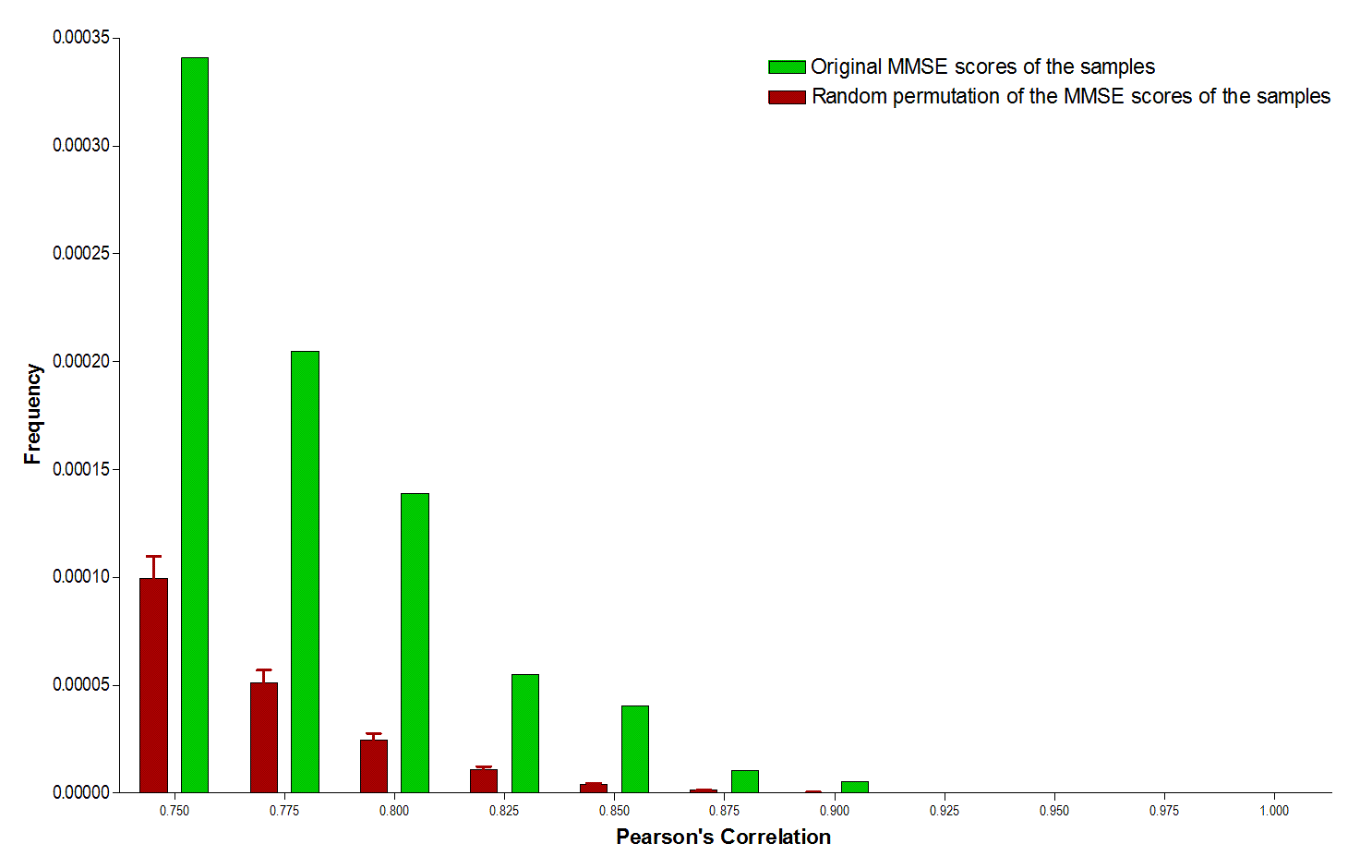
**

The correlation cut-off is chosen according to the MMSE cluster in Table 3 of the manuscript. Test phenotype: MMSE, correlation metric: Pearson’s correlation coefficient and data set: 941,885 ratio metafeatures data set.

We permuted (rearranged) the phenotype (for instance, MMSE) samples using “random_shuffle” function of STL (Standard Template Library) and computed the Pearson’s correlation of each metafeatures in the “941,885 ratio metafeatures data set” with the permuted samples. We conducted the test for a total of *i*=1,000 iterations and visualized the average outcome tests. The method produces a far greater number of highly correlated metafeatures than would be expected by chance alone (i.e. when randomly permuting the phenotype labels). This figure also facilitates an estimation of the false positive rate at different correlation coefficient thresholds.
