# Supplementary material for: Unveiling Clusters of RNA Transcript Pairs Associated with Markers of Alzheimer’s Disease Progression
Source: PLoS One. 2012 Sep 21;7(9):e45535. doi: 10.1371/journal.pone.0045535 (PMC3448659; doi:10.1371/journal.pone.0045535)
Supplement: Figure S6 — Counter example for the correlation of probe set pairs. (DOC) [file pone.0045535.s006.doc]

**Figure S6. Counter example for the correlation of probe set pairs.**

|  |  |  |
| --- | --- | --- |
|  |  |  |
|  |  |  |

Not all pairs of probe sets provide a better correlation than either probe set alone. This does not seem to be a universal phenomenon – instead it may be the case that two probe sets that have high correlations individually may end up having, when taken together as a ratio metafeature, a lower correlation.
