# Supplementary material for: Unveiling Clusters of RNA Transcript Pairs Associated with Markers of Alzheimer’s Disease Progression
Source: PLoS One. 2012 Sep 21;7(9):e45535. doi: 10.1371/journal.pone.0045535 (PMC3448659; doi:10.1371/journal.pone.0045535)
Supplement: Table S1 — Analysis of the 941,885 ratio metafeatures clustered with the MMSE score. (DOC) [file pone.0045535.s007.doc]

**Table S1. Analysis of the 941,885 ratio metafeatures clustered with the MMSE score.**

| **Metafeature (Gene Symbol. Probe Set ID)** | **Correlation of individual probe significant (p < 0.05)?** | |
| --- | --- | --- |
|  | **Probe Set 1** | **Probe Set 2** |
| **[PPIA**.212661_x_at/C3orf60.209177_at] | YES | NO |
| [PIGO.209998_at/**CASK**.211208_s_at] | NO | YES |
| [PURA.213806_at/AJ225093.211835_at] | NO | YES |
| [**PRKCB1**.209685_s_at/**PTEN**.222176_at] | NO | NO |
| [**FOXO1**.202724_s_at/**ACACA**.212186_at] | YES | NO |
| [**CASK**.211208_s_at/**PTEN**.222176_at] | YES | NO |
| [RPL23A.203012_x_at/**ATP5C1**.213366_x_at] | NO | YES |
| [**CASK**.211208_s_at/**ATP5C1**.213366_x_at] | YES | YES |
| [**ACACA**.212186_at/**LDHA**.200650_s_at] | NO | YES |
| [**PRKAR2B**.203680_at/**ACACA**.212186_at] | NO | NO |
| [COX6A1.200925_at/**ATP5C1**.213366_x_at] | NO | YES |
| [RPL23A.203012_x_at/**PPIA**.211765_x_at] | NO | YES |
| [ACTN1.208636_at/VCL.200930_s_at] | NO | NO |
| [**ITGB8**.205816_at/**TTN**.208195_at] | YES | YES |
| [**ITGB8**.205816_at/PTN.209466_x_at] | YES | YES |
| [**CPT2**.204264_at/**ATP5C1**.213366_x_at] | YES | YES |
| [**TTN**.208195_at/**PRKCB1**.209685_s_at] | YES | NO |
| [SDC1.201287_s_at/**DDX1**.201241_at] | YES | YES |
| [**TTN**.208195_at/**NEFL**.221805_at] | YES | YES |
| [ZNF34.219801_at/**FXYD6**.217897_at] | YES | YES |
| [**AL359052**.214927_at/**ATP5C1**.205711_x_at] | YES | YES |
| [NUCKS1.217802_s_at/**PPIA**.212661_x_at] | NO | YES |
| [**TCF7L2**.212761_at/ACTN1.208636_at] | YES | NO |
| [**TTN**.208195_at/**VSNL1**.203798_s_at] | YES | YES |
| [**CASK**.211208_s_at/**PTN**.209466_x_at] | YES | YES |
| [NM_024849.220531_at/MRPL16.217980_s_at] | YES | YES |
| [AJ225093.211835_at/**ATP5C1**.205711_x_at] | YES | YES |
| [**C10orf76**.55662_at/**PPIA**.212661_x_at] | YES | YES |
| [**ITGB8**.205816_at/SDC1.201287_s_at] | YES | YES |
| [**ITGB8**.205816_at/**PPIA**.212661_x_at] | YES | YES |
| [**CASK**.211208_s_at/**PPIA**.211765_x_at] | YES | YES |
| [TUG1.222244_s_at/**SCFD1**.215548_s_at] | YES | YES |

Metafeatures are ordered by Spearman’s rank correlation with MMSE score. Genes in boldface indicate that they were previously discussed in [1] and genes with underlined boldface represent the cases for which the gene has been discussed in the context of AD in the published literature.

[1] Gomez Ravetti M, Rosso OA, Berretta R, Moscato P (2010) Uncovering molecular biomarkers that correlate cognitive decline with the changes of hippocampus' gene expression profiles in Alzheimer's disease. PLoS One 5: e10153.
