# Supplementary material for: Unveiling Clusters of RNA Transcript Pairs Associated with Markers of Alzheimer’s Disease Progression
Source: PLoS One. 2012 Sep 21;7(9):e45535. doi: 10.1371/journal.pone.0045535 (PMC3448659; doi:10.1371/journal.pone.0045535)
Supplement: Table S2 — False discovery rate (FDR) in 1,372-probe AD signature data set. (DOC) [file pone.0045535.s008.doc]

**Table S2. False discovery rate (FDR) in 1372-probe AD signature data set.**

| Correlation Phenotype | Frequency | |
| --- | --- | --- |
|  | Correlations which are greater than the lowest positive correlation in the MMSE cluster (0.590462) | Correlations which are lower than the lowest negative correlation in the MMSE cluster (-0.48268) |
| MMSE scores of the samples | 17 | 125 |
| Random permutations (1,000 ) of the MMSE scores of the samples | 2.106 | 6.208 |
| FDR | 12.39% | 4.97% |

FDRs are calculated as follows,

$FDR=\frac{\text{Frequency of the correlations over the threshold (random perm of the MMSE scores)}}{\text{Frequency of the correlations over the threshold (MMSE scores of the samples)}}*100$%
