# Supplementary material for: Unveiling Clusters of RNA Transcript Pairs Associated with Markers of Alzheimer’s Disease Progression
Source: PLoS One. 2012 Sep 21;7(9):e45535. doi: 10.1371/journal.pone.0045535 (PMC3448659; doi:10.1371/journal.pone.0045535)
Supplement: Table S3 — False discovery rate (FDR) 941,885 ratio metafeatures data set. (DOC) [file pone.0045535.s009.doc]

**Table S3.** **False discovery rate (FDR) 941885-AD ratio metafeatures data set**

| Correlation Phenotype | Frequency | |
| --- | --- | --- |
|  | Correlations which are greater than the lowest positive correlation in the MMSE cluster (0.673877) | Correlations which are lower than the lowest negative correlation in the MMSE cluster (-0.68675) |
| MMSE scores of the samples | 1,291 | 2,451 |
| Random permutations (1,000) of the MMSE scores of the samples | 221.962 | 180.181 |
| FDR | 17.13% | 7.35% |

FDRs are calculated as follows,

$FDR=\frac{\text{Frequency of the correlations over the threshold (random perm of the MMSE scores)}}{\text{Frequency of the correlations over the threshold (MMSE scores of the samples)}}*100$%
