# Supplementary material for: Unveiling Clusters of RNA Transcript Pairs Associated with Markers of Alzheimer’s Disease Progression
Source: PLoS One. 2012 Sep 21;7(9):e45535. doi: 10.1371/journal.pone.0045535 (PMC3448659; doi:10.1371/journal.pone.0045535)
Supplement: Table S4 — Pair-wise comparisons of overlap between progression marker clustering outcomes in the 941,885 metafeatures data set. (DOC) [file pone.0045535.s010.doc]

**Table S4. Pair-wise comparisons of overlap between progression marker clustering outcomes in the 941,885 metafeatures data set.**

|  | **MMSE** | **NFT** | **Braak’s Staging** | **JSDcontrol** | **JSDsevere** |
| --- | --- | --- | --- | --- | --- |
| **MMSE** |  | ITGB8 | PTN | PPIA  ATP5C1  LDHA  DDX1  SCFD1 | PTEN  PRKCB1  CPT2 |
| **NFT** | ITGB8 |  | $\varphi$ | ICA1 | $\varphi$ |
| **Braak’s** | PTN | $\varphi$ |  | PTN | $\varphi$ |
| **JSDcontrol** | PPIA  ATP5C1  LDHA  DDX1  SCFD1 | ICA1 | PTN |  | $\varphi$ |
| **JSDsevere** | PTEN  PRKCB1  CPT2 | $\varphi$ | $\varphi$ | $\varphi$ |  |

Clustering of the 941,885 metafeatures data set identified metafeatures that correlated with each of the different progression markers. In several cases, the metafeatures clustered with one progression marker contained probe sets that also contributed to metafeatures clustered with a different progression marker. This table shows which transcripts were targeted by probe sets that clustered with two different progression markers.
