# Supplementary material for: Unveiling Clusters of RNA Transcript Pairs Associated with Markers of Alzheimer’s Disease Progression
Source: PLoS One. 2012 Sep 21;7(9):e45535. doi: 10.1371/journal.pone.0045535 (PMC3448659; doi:10.1371/journal.pone.0045535)
Supplement: Table S5 — Pair-wise comparisons of overlap between progression marker clustering outcomes in the 3,763,403 metafeatures data set. (DOC) [file pone.0045535.s011.doc]

**Table S5. Pair-wise comparisons of overlap between progression marker clustering outcomes in the 3,763,403 metafeatures data set**

|  | **MMSE** | **NFT** | **Braak’s Staging** | **JSDcontrol** | **JSDsevere** |
| --- | --- | --- | --- | --- | --- |
| **MMSE** |  | PPIA  TTN  C10orf76 | ATP5C1 | ICA1  RBM19  LDHA  COX6A1 | CPT2  PTEN  **VSNL1**  COX6A1 |
| **NFT** | PPIA  TTN  C10orf76 |  | $\varphi$ | MMP11  ICA1 | $\varphi$ |
| **Braak’s** | ATP5C1 | $\varphi$ |  | $\varphi$ | $\varphi$ |
| **JSDcontrol** | ICA1  RBM19  LDHA  COX6A1 | MMP11  ICA1 | $\varphi$ |  | GABRQ  COX6A1 |
| **JSDsevere** | CPT2  PTEN  **VSNL1**  COX6A1 | $\varphi$ | $\varphi$ | COX6A1  GABRQ |  |

Clustering of the 3,763,403 metafeatures data set identified metafeatures that correlated with each of the different progression markers. In several cases, the metafeatures clustered with one progression marker contained probe sets that also contributed to metafeatures clustered with a different progression marker. This table shows which transcripts were targeted by probe sets that clustered with two different progression markers. VSNL1 is highlighted in boldface as a recent study by Tarawneh et al. [1] indicate that VSNL1 abundance in CSF, and the ratio of its abundance with that of *Aβ42* can offer a diagnostic tool to detect early AD and *“predict future cognitive impairment in cognitively normal individuals similarly to tau and tau/Aβ42, respectively”.*

[1] Tarawneh R, D'Angelo G, Macy E, Xiong C, Carter D, et al. (2011) Visinin-like protein-1: diagnostic and prognostic biomarker in Alzheimer disease. Ann Neurol 70: 274-285.
