# Supplementary material for: Unveiling Clusters of RNA Transcript Pairs Associated with Markers of Alzheimer’s Disease Progression
Source: PLoS One. 2012 Sep 21;7(9):e45535. doi: 10.1371/journal.pone.0045535 (PMC3448659; doi:10.1371/journal.pone.0045535)
Supplement: File S4 — Implementation of the proposed clustering method. (PDF) [file pone.0045535.s015.pdf]

Supporting Information File S4

The computational tests were performed on the following hardware setup. Four NVIDIA Tesla C2050 GPU cards installed on a Xenon Nitro T5 Supermicro server that has Dual Xeon 5620 2.4GHz processors, 32GB of 1066 MHz DDR3 RAM and 800GB of Local Hard Disk. The programs were written in C++, STXXL [5], and CUDA and compiled using the `g++` 4.4.4 and `nvcc` compiler on a Linux OS, Kernel version 2.6.9.

Implementation of the Clustering Method

We re-implemented a graph-based clustering algorithm known as MSTkNN, proposed in [8] to tackle the task of clustering large-scale biological networks. The detailed method is presented in [2]. To implement this method we computed the k-Nearest Neighbour (kNN) graph, minimum spanning tree (MST) and connected components (CCs) as follows:

Computation of kNN Graphs using GPUs. The basic kNN search technique is simple and straightforward and one can use an exhaustive search technique (also known as brute force approach) to find the nearest neighbours of a point. However, the actual computation of the distances and the nearest neighbours for large-scale instances requires a large number of computations. Several sequential approaches were proposed in [4, 3] to tackle this problem when an approximate solution is sufficient. Interestingly, the basic brute force approach that gives the exact solution to this problem is highly parallelizable as the nearest neighbours of each query point can be computed and searched independently. This particular feature influenced us to create a GPU based parallel implementation for tackling this problem. Our approach is scalable and independent of the instance size as we only work with a part of the data and computation, at a time. The implementation is designed to work with NVIDIAs Compute Unified Device Architecture (CUDA) enabled GPUs, but the approach is adaptable to other architectures as well.

The most common problems with the existing GPU-based brute force kNN algorithms are two-fold, firstly, they can only work if all the distances between query and reference points, i.e., the distance matrix, can fit into GPUs in-memory; secondly, they assume that the value of k is relatively small in comparison with the instance size and thus, they store the kNNs list and related distances in shared memory, which is fast but very small in size. We overcome the first problem by dividing the complete distance matrix into several sub-matrices (chunks) and subsequently we compute and identify the partial kNNs from these sub-matrices. We do not store the complete distance matrix at any stage of the computation, thus the method is independent of the distance matrix size. The second problem is handled by storing the kNNs and related values into GPU's global memory which is much larger than the shared memory, assuming that the value of k will always be less than the predefined chunk size. The whole approach slows down the kNN search slightly but scales the method to its limit

on the device. Additionally, we get a portion of the kNN graph whenever the device finishes computing a part of the distance matrix and thus, for larger instances, the kNN graph can also be stored into an external memory, whenever a portion of the graph is finished. Our method is based on two different CUDA kernels that are executed one after another. The purpose of the first kernel is to compute a distance matrix and the second kernel will produce a kNN graph from the distance matrix . If the distance matrix can completely fits into GPUs in-memory, we consider the whole distance matrix as a single *chunk* and we execute the distance kernel to produce a distance matrix and then, we invoke the kNN kernel to produce the kNN graph, given the value of k. On the contrary, if the distance matrix is too large to fit into the devices in-memory, we break down the distance matrix into several chunks (sub-matrices), the size of the chunk is given as an input parameter. We assume that all the chunks that share the same rows in a distance matrix are in a same *split*. When all the chunks in a split are executed, we get a partial kNN graph. Furthermore, if we have multiple GPUs, we can again subdivide the splits into several segments, each of which can be handled in parallel by different GPUs. Same as previously, when all the splits in a segment are executed, we get a partial kNN graph and after the execution of all the segments, we get the complete the kNN graph. In Figure 1, we have demonstrated the basic working principle of our proposed method.

Minimum Spanning Tree Computation using External Memory Algorithms. In this work, we have adapted an external memory algorithm for computing the minimum spanning trees (EM MST) introduced by Dementiev et al. in [6] that is considerably simpler than previously known external memory algorithms and needs a factor of at least four less I/Os for realistic inputs. The I/O complexity of this algorithm is $\mathcal{O}(\text{sort}(m) \cdot \log(n/M))$, where n, m and M are the number of nodes, number of edges and number of nodes fit in internal memory respectively. The basic goal of the EM MST algorithm by Dementiev et al. [6] is to reduce the number of nodes of the original graph G until the union-find data structure ([12]) of the remaining nodes (graph $G^{'}$) fits in internal memory so that the semi-external Kruskal algorithm can be applied to $G^{'}$. The external implementation of EM MST algorithm[1] uses STXXL[2] [5], a sweeping algorithm using external sorting [7] and external priority queues [9]. The implementation can solve instances with up to 2^{32} (i.e., more than 4 billions) nodes.

External Memory Connected Components. A connected component is the edge set induced by a maximal set of vertices such that each pair of vertices is connected by a path in G. The external minimum spanning tree algorithm presented in [6] was adopted by Schultes [10] in order to determine the spanning forests of an unweighted graph. Furthermore, the author has also presented an

[1]See, http://algo2.iti.kit.edu/schultes/emmst/

[2]Available at http://stxxl.sourceforge.net/

implementation of the connected components(CC) algorithm[3] by adapting the algorithms in [11] and [1]. The results in [10] show that computation of spanning forests or the connected components of an unweighted graph is about twice as fast as the computation of a minimum spanning tree of an weighted graph. We have adapted the implementation by Schultes [10] to identify the connected components from the disconnected minimum spanning tree.

The MSTkNN algorithm in [8] stores each connected component in a separate in-memory matrix (sub-matrix) that consumes an amount of internal memory and finally large number of clusters, this implementation becomes incomputable. In contrast, we store the connected components (clusters) in external memory and only keep the list of the components in computers in-memory. This eliminates the excessive use of the in-memory even when there are a large number of components or clusters. In addition to these modifications, we tuned the implementations of the adapted algorithms ([6] and [11]) for better performance with comparatively denser graphs. As a result, our implementation is faster (due to the algorithmic enhancement), memory efficient (due to the EM implementation) and much more scalable (due to parallel/distributed pre-processing) than the one in [8]. This enabled us to cluster larger networks than previously possible (see details in [2]).

References

[1] Abello, J., Buchsbaum, A. L., Westbrook, J. R., 1998. A functional approach to external graph algorithms. In: Algorithmica. Springer-Verlag, pp. 332–343.

[2] Arefin, A. S., Inostroza-Ponta, M., Mathieson, L., Berretta, R., Moscato, P., 2011. Clustering nodes in large-scale biological networks using external memory algorithms. In: Xiang, Y., Cuzzocrea, A., Hobbs, M., Zhou, W. (Eds.), ICA3PP (2). Vol. 7017 of Lecture Notes in Computer Science. Springer, pp. 375–386.

[3] Arya, S., Mount, D. M., Netanyahu, N. S., Silverman, R., Wu, A. Y., nov 1998. An optimal algorithm for approximate nearest neighbor searching fixed dimensions. J. ACM 45 (6), 891–923.
URL `http://dx.doi.org/10.1145/293347.293348`

[4] Bentley, J. L., sep 1975. Multidimensional binary search trees used for associative searching. Commun. ACM 18 (9), 509–517.
URL `http://dx.doi.org/10.1145/361002.361007`

[5] Dementiev, R., Kettner, L., Sanders, P., May 2008. Stxxl: standard template library for xxl data sets. Softw. Pract. Exper. 38, 589–637.
URL `http://dl.acm.org/citation.cfm?id=1361730.1361733`

[3] See, http://algo2.iti.kit.edu/dementiev/files/cc.tgz

[6] Dementiev, R., S, P., Schultes, D., Sibeyn, J., 2004. Engineering an external memory minimum spanning tree algorithm. In: In Proc. 3rd IFIP Intl. Conf. on Theoretical Computer Science. Kluwer, pp. 195–208.

[7] Dementiev, R., Sanders, P., 2003. Asynchronous parallel disk sorting. In: SPAA '03: Proceedings of the fifteenth annual ACM symposium on Parallel algorithms and architectures. ACM, New York, NY, USA, pp. 138–148.

[8] Inostroza-Ponta, M., 2008. An integrated and scalable approach based on combinatorial optimization techniques for the analysis of microarray data. Ph.D. thesis, School of Electrical Engineering and Computer Science, The University of Newcastle, Australia.

[9] Sanders, P., 2000. Fast priority queues for cached memory. J. Exp. Algorithmics 5, 7.

[10] Schultes, D., September 2004. External memory spanning forests and. http://algo2.iti.kit.edu/dementiev/files/cc.pdf.

[11] Sibeyn, J. F., 2004. External connected components. In: Hagerup, T., Katajainen, J. (Eds.), SWAT. Vol. 3111 of Lecture Notes in Computer Science. Springer, pp. 468–479.

[12] Tarjan, R. E., 1975. Efficiency of a good but not linear set union algorithm. J. ACM 22 (2), 215–225.

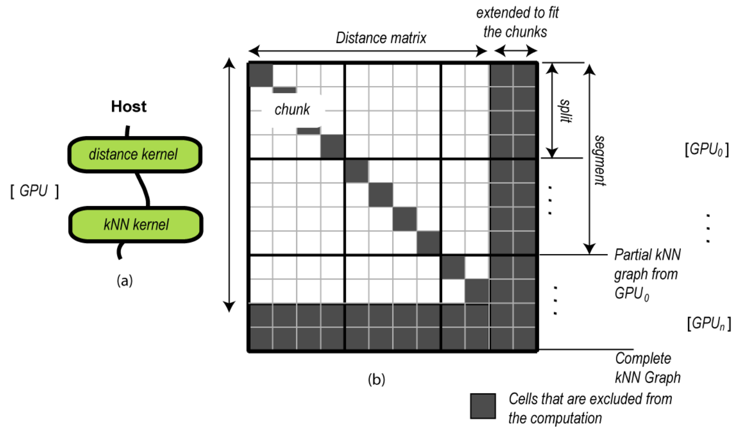

Figure 1: (a) Figure shows two kernels that execute sequentially, the first one create the distance matrix and the second one identifies the nearest neighbour graph. Figure (b) shows the proposed method of computing kNN using chunk, split and segment in the matrix.
